# Loss of genome maintenance is linked to mTOR complex 1 signaling and accelerates podocyte damage

**DOI:** 10.1172/jci.insight.172370

**Published:** 2025-05-20

**Authors:** Fabian Braun, Amrei M. Mandel, Linda Blomberg, Milagros N. Wong, Georgia Chatzinikolaou, David H. Meyer, Anna Reinelt, Viji Nair, Roman Akbar-Haase, Phillip J. McCown, Fabian Haas, He Chen, Mahdieh Rahmatollahi, Damian Fermin, Robin Ebbestad, Gisela G. Slaats, Tillmann Bork, Christoph Schell, Sybille Koehler, Paul T. Brinkkoetter, Maja T. Lindenmeyer, Clemens D. Cohen, Martin Kann, David Unnersjö-Jess, Wilhelm Bloch, Matthew G. Sampson, Martijn E.T. Dollé, Victor G. Puelles, Matthias Kretzler, George A. Garinis, Tobias B. Huber, Bernhard Schermer, Thomas Benzing, Björn Schumacher, Christine E. Kurschat

**Affiliations:** 1 Department II of Internal Medicine and Center for Molecular Medicine Cologne, University of Cologne, Faculty of Medicine and University Hospital Cologne, Cologne, Germany.; 2 III. Department of Medicine,; 3 Hamburg Center for Kidney Health (HCKH), and; 4 Martin Zeitz Center for Rare Diseases, University Medical Center Hamburg-Eppendorf, Hamburg, Germany.; 5 Cologne Excellence Cluster on Cellular Stress Responses in Aging-Associated Diseases (CECAD), University of Cologne, Faculty of Medicine and University Hospital Cologne, Cologne, Germany.; 6 Research Center On Rare Kidney Diseases (RECORD), University Hospital Erlangen, Erlangen, Germany.; 7 Department of Clinical Medicine, Aarhus University, Aarhus, Denmark.; 8 Department of Pathology, Aarhus University Hospital, Aarhus, Denmark.; 9 Institute of Molecular Biology and Biotechnology, Foundation for Research and Technology-Hellas, Heraklion, Crete, Greece.; 10 Institute for Genome Stability in Aging and Disease, Medical Faculty, University and University Hospital of Cologne, Germany.; 11 Division of Nephrology, Department of Internal Medicine, and; 12 Department of Pediatrics-Nephrology, University of Michigan, Ann Arbor, Michigan, USA.; 13 Science for Life Laboratory, Department of Women’s and Children’s Health, Karolinska Institutet, Stockholm, Sweden.; 14 Department of Medicine IV, Faculty of Medicine; and; 15 Institute of Surgical Pathology, Faculty of Medicine, University of Freiburg, Freiburg, Germany.; 16 Nephrological Center, Medical Clinic and Polyclinic IV, University of Munich, Munich, Germany.; 17 Science for Life Laboratory, Department of Applied Physics, Royal Institute of Technology, Solna, Sweden.; 18 Division of Renal Medicine, Department of Clinical Sciences, Intervention and Technology (CLINTEC), Karolinska Institute, Stockholm, Sweden.; 19 Department of Molecular and Cellular Sport Medicine, German Sport University Cologne, Cologne, Germany.; 20 Division of Nephrology, Boston Children’s Hospital, Boston, Massachusetts, USA.; 21 Harvard Medical School, Boston, Massachusetts, USA.; 22 Broad Institute, Cambridge, Massachusetts, USA.; 23 National Institute for Public Health and the Environment, Bilthoven, Netherlands.; 24 Department of Biology, University of Crete, Heraklion, Crete, Greece.; 25 Systems Biology of Ageing Cologne (SyBaCol), University of Cologne, Cologne, Germany.

**Keywords:** Aging, Cell biology, Nephrology, Chronic kidney disease, DNA repair

## Abstract

DNA repair is essential for preserving genome integrity. Podocytes, postmitotic epithelial cells of the kidney filtration unit, bear limited regenerative capacity, yet their survival is indispensable for kidney health. Podocyte loss is a hallmark of the aging process and of many diseases, but the underlying factors remain unclear. We investigated the consequences of DNA damage in a podocyte-specific knockout mouse model for DNA excision repair protein *Ercc1* and in cultured podocytes under genomic stress. Furthermore, we characterized DNA damage-related alterations in mouse and human renal tissue of different ages and patients with minimal change disease and focal segmental glomerulosclerosis. *Ercc1* knockout resulted in accumulation of DNA damage and ensuing albuminuria and kidney disease. Podocytes reacted to genomic stress by activating mTOR complex 1 (mTORC1) signaling in vitro and in vivo. This was abrogated by inhibiting DNA damage signaling through DNA-dependent protein kinase (DNA-PK) and ataxia teleangiectasia mutated (ATM) kinases, and inhibition of mTORC1 modulated the development of glomerulosclerosis. Perturbed DNA repair gene expression and genomic stress in podocytes were also detected in focal segmental glomerulosclerosis. Beyond that, DNA damage signaling occurred in podocytes of healthy aging mice and humans. We provide evidence that genome maintenance in podocytes is linked to the mTORC1 pathway and is involved in the aging process as well as the development of glomerulosclerosis.

## Abstract 2

DNA damage in podocytes leads to accelerated cell loss and sclerosis. This is linked to mTORC1 signaling through DNA damage signaling and plays a role in glomerular diseases.

## Introduction

Most cells of the body are constantly subjected to various DNA-damaging agents (1, 2). Therefore, cells depend on numerous DNA repair mechanisms to counteract transcription stress (3). Mutations in DNA repair genes result in a variety of pathologies ranging from cancer to progeroid syndromes (4, 5). The specific importance of genome maintenance in cells with limited regenerative capacity is demonstrated by the prevalence of neurodegeneration as a hallmark of DNA repair deficiency syndromes (6).

Glomerular podocytes are terminally differentiated, postmitotic cells with little to no replacement capacity after development (7). As an integral part of the primary filtration unit of the kidney (8), podocyte depletion is a leading cause of chronic kidney disease due to diabetes, hypertension, and other glomerulopathies, with ensuing loss of protein into the urine (9) and progressive renal insufficiency. A milder phenotype without overt proteinuria is present in aged kidneys, with globally sclerosed glomeruli due to decreasing numbers of podocytes with age (10). The precise pathomechanisms leading to podocyte depletion, however, are incompletely understood. Protecting this finite number of cells is, therefore, an important therapeutic goal (11).

Lately, studies have indicated the importance of genome maintenance for renal health (12, 13). Mutations in the kinase endopeptidase and other proteins of small size (KEOPS) complex genes caused proteinuria and induced DNA damage response (DDR) in vitro (14). Likewise, glomerular DNA damage was found to be associated with declining kidney function (15), and cells isolated from the urine of patients with diabetes and hypertension showed increased levels of DNA strand breaks (16). Furthermore, the glomeruli of a progeroid mouse model were identified to share the expression profile of aged glomeruli, indicating a role of DNA damage repair in glomerular aging (17).

Several studies have proposed an interplay between DNA damage signaling and the mTOR pathway, with factors induced by DNA damage exhibiting repressive effects on mTOR complex 1 (mTORC1) (18–20) and increased mTORC1 activity leading to transcription stress (21–23). This is of particular interest for podocytes, as they are highly dependent on a rigorous control of mTOR activity. While mTORC1-driven hypertrophy is a protective response upon podocyte depletion (10, 24, 25), mTORC1 overactivation drives pathologic hyperproliferation and sclerosis (26, 27). In line with these findings, side effects of pharmacological mTORC1 inhibition entail proteinuria and glomerular scarring (28–34).

Our group has proposed a role of DNA damage in glomerular aging by investigating glomeruli of the progeroid *Ercc1*^–/Δ^ mouse (17). We followed up on these analyses and detected a newly discovered hallmark of aging — the shift toward transcription of smaller RNAs resulting from transcriptional stalling (35). To further address the involvement of genomic stress in podocyte damage and accelerated aging, we analyzed a podocyte-specific DNA excision repair protein *Ercc1*-knockout mouse model.

## Results

### DNA damage repair is essential for podocyte health.

**Figure 1:**
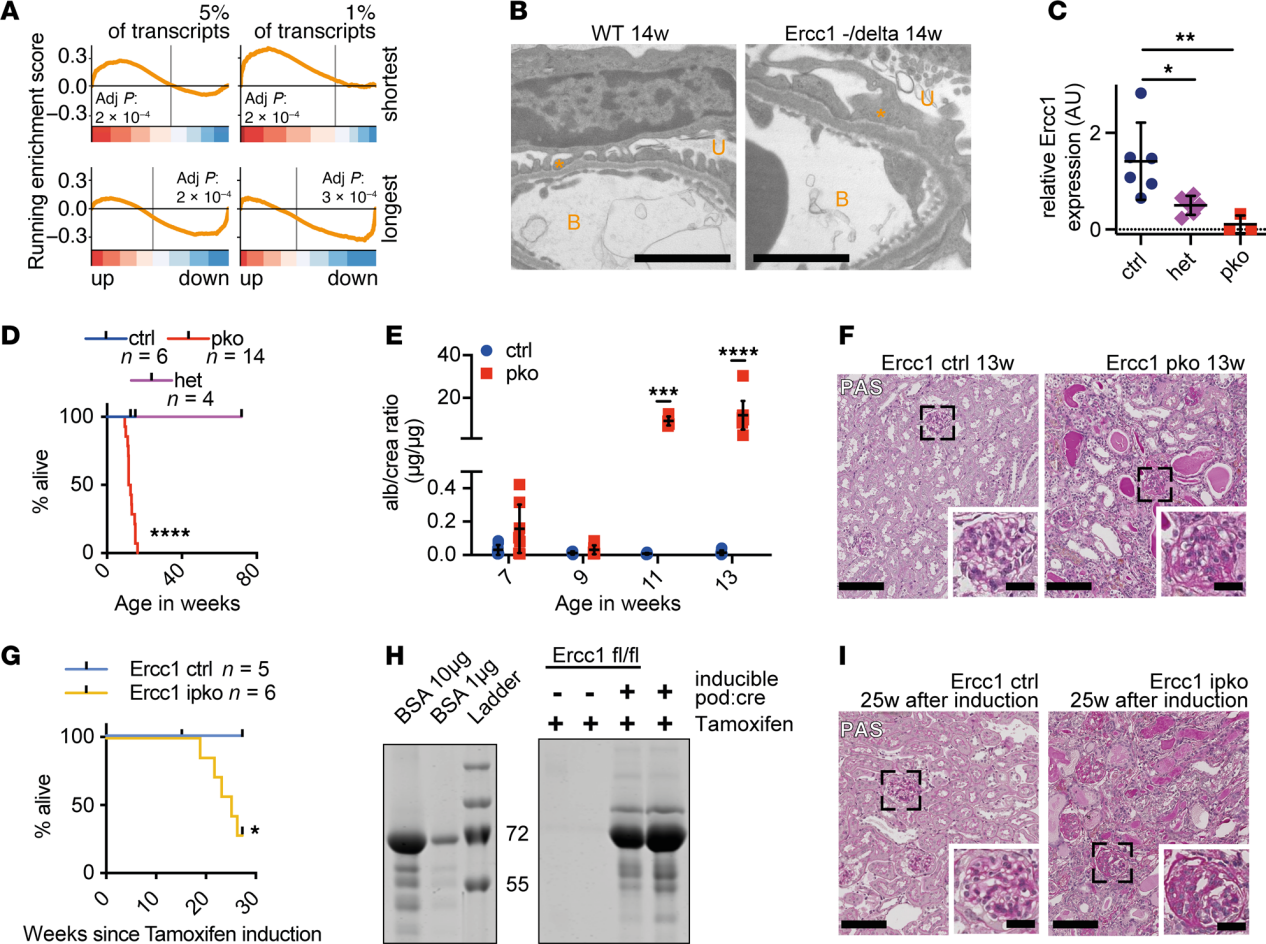
Podocyte-specific *Ercc1* deletion causes glomerulosclerosis. (**A**) Gene set enrichment analysis (GSEA) of gene classes according to transcript length. Shown are the shortest 5% (top left), shortest 1% (top right), longest 5% (bottom left), and longest 1% (bottom right) of genes. Bottom color-coded panel shows log_2_ fold-changes of microarray data in ranked order. Top panels show running enrichment score as orange line. Smallest 1% (normalized enrichment score [NES] of 2.01) and 5% (NES of 1.37) of genes are significantly enriched in upregulated genes, while longest 1% (NES of –1.69) and 5% (NES of –1.42) of genes are significantly enriched in downregulated genes in the comparison of *Ercc1*^–/Δ^ 14 weeks versus WT 14 weeks (*n* = 4). (**B**) Representative electron microscopy image of 14-week-old WT and *Ercc1*^–/Δ^ glomerular filtration barrier, scale bar indicating 2 μm. B, blood side; U, urinary side; asterisk, foot process (*n* = 4). (**C**) Quantitative PCR (qPCR) analysis for *Ercc1* in FACS-sorted podocytes of *Ercc1* ctrl, WT/pko (het), or pko mice (1-way ANOVA with Tukey’s multiple comparisons test, *n* = 3–6). (**D**) Survival of *Ercc1* ctrl, WT/pko (het), and pko mice (Mantel-Cox test, *n* = 4–14). (**E**) Urinary albumin/creatinine analysis of *Ercc1* ctrl and pko mice (2-way ANOVA with Šídák’s multiple comparisons test, *n* = 4–9). (**F**) Representative periodic acid–Schiff (PAS) staining of 13-week-old *Ercc1* ctrl and pko mice, scale bars: 100 μm in overview, 30 μm in zoom (*n* = 6). (**G**) Kaplan-Meier curve depicting survival of *Ercc1* ctrl and ipko mice (Mantel-Cox test, *n* = 5–6). (**H**) Representative Coomassie staining of *Ercc1* ctrl and ipko urine 18 weeks after tamoxifen induction; bovine serum albumin was loaded as reference (*n* = 6). Values are in kilodaltons. (**I**) Representative PAS staining of *Ercc1* ctrl and ipko mice 25 weeks after induction with tamoxifen (*n* = 6). Scale bar as in **F**. Scatterplots indicate mean plus 95% confidence interval. *P ≤ 0.05, **P ≤ 0.01, ***P ≤ 0.001, ****P ≤ 0.0001.

A well-established mouse model to induce DNA damage in vivo is the deletion of Ercc1, a cofactor for endonuclease Ercc4 (36–38). Our group has proposed a role of DNA damage in glomerular aging by investigating glomeruli of the *Ercc1*^–/Δ^ mouse (39), a model with whole-body disruption of *Ercc1* on one allele and a truncated form of *Ercc1* on the second allele, leading to a hypomorphic variant with minimal residual Ercc1 activity. We previously showed that the expression profile of *Ercc1*^–/Δ^ glomeruli shared similarities with the profile of aged glomeruli. Recently, a shift toward small RNA transcripts as a novel hallmark of aging was described (35); thus, we compared the length of RNA transcripts in prematurely aged 14-week-old *Ercc1*^–/Δ^ glomeruli with WT glomeruli of the same age. RNA expression showed an upregulation of the 5% and 1% shortest and downregulation of the 5% and 1% longest transcripts (Figure 1A and Supplemental Figure 1; supplemental material available online with this article; https://doi.org/10.1172/jci.insight.172370DS1), validating transcriptional stalling as a hallmark of the aging process in *Ercc1*-deficient glomeruli (35). This glomerular phenotype coincided with the development of foot process effacement, a characteristic feature of podocyte damage, detected in electron microscopy (Figure 1B), pointing toward DNA damage repair as an essential component of glomerular aging and podocyte health. To specifically investigate the role of DNA damage repair and aging on podocytes, we generated a constitutive podocyte-specific knockout of *Ercc1* (pko) using the cre-*loxP* system in mice of mixed FVB/CD1 background (40) (Figure 1C and Supplemental Figure 2A), expecting a phenotype of accelerated podocyte aging. Mice carrying the pko had a dramatically decreased lifespan of 15–20 weeks, while Cre-negative animals (ctrl) and Cre-positive animals heterozygous for the floxed *Ercc1* allele (het) remained without overt abnormalities for up to 72 weeks (Figure 1D and Supplemental Figure 2, E and F). This decreased lifespan was accompanied by significant albuminuria and severe kidney failure represented by elevated serum creatinine and urea levels, starting at week 11 (Figure 1E and Supplemental Figure 2, B and C). At week 13, *Ercc1*-pko mice developed severe generalized renal damage, including glomerulosclerosis, interstitial fibrosis, and tubular atrophy with protein casts (Figure 1F and Supplemental Figure 2D).

Similar results could be obtained in a tamoxifen-inducible podocyte-specific knockout (ipko) of *Ercc1* (Figure 1, G–I, and Supplemental Figure 2G), indicating that this phenotype was not dependent on developmental abnormalities in the absence of Ercc1.

### DNA damage accumulation in podocytes triggers cellular stress and podocyte loss.

**Figure 2:**
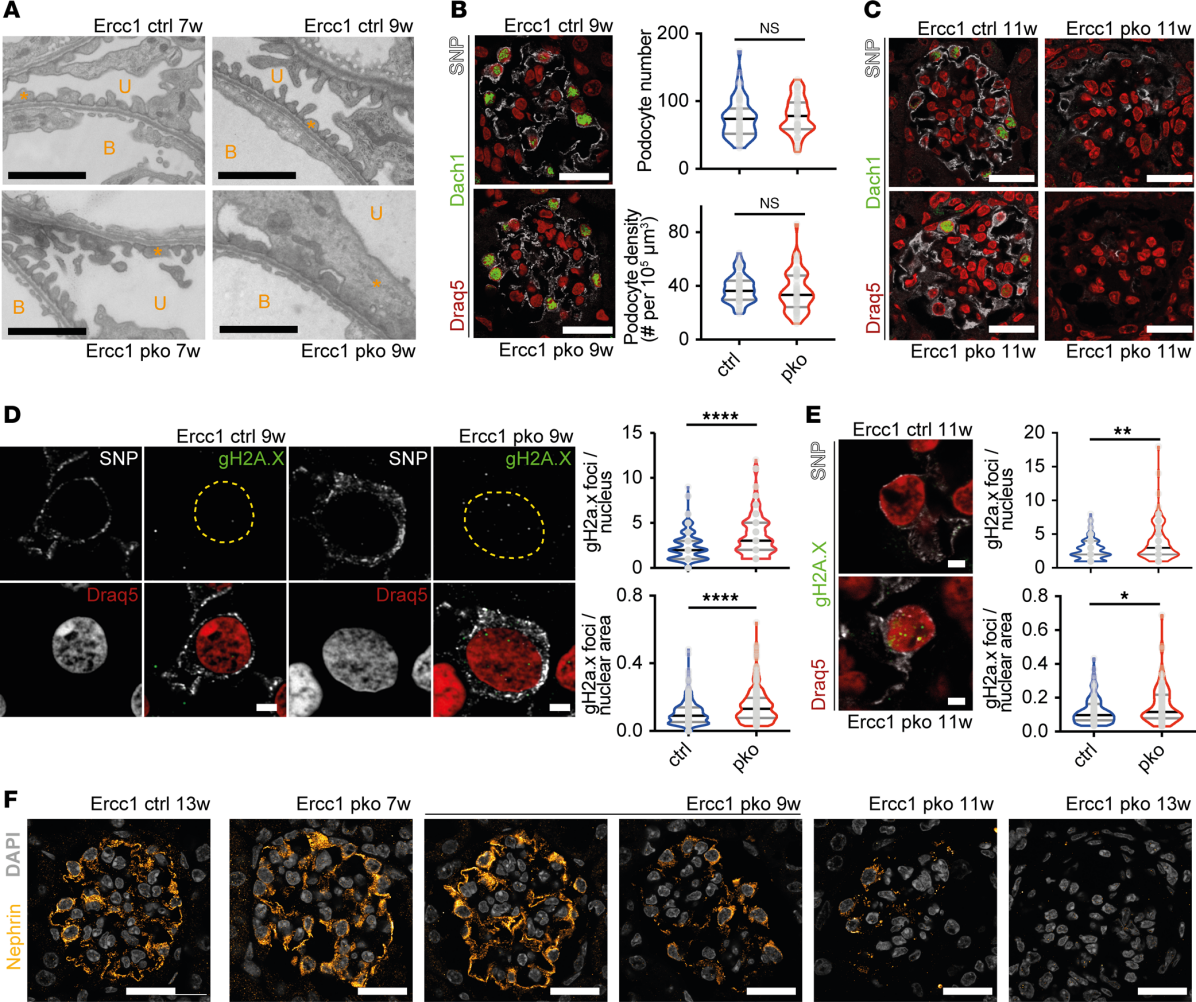
The podocyte-specific constitutive knockout of *Ercc1* leads to foot process effacement and podocyte loss accompanied by accumulation of DNA damage. (**A**) Representative electron microscopy image of 7- and 9-week-old *Ercc1* ctrl and pko glomerular filtration barrier, scale bar: 2 μm. B, blood side; U, urinary side; asterisk: foot process (*n* = 3). (**B**) Representative immunofluorescence staining of synaptopodin (SNP, gray), dachshund family transcription factor 1 (Dach1, green) (75), and nuclei (Draq5, red) in sections of 9-week-old *Ercc1* ctrl and pko kidneys, with quantification of podocyte number and density, scale bar indicating 10 μm (unpaired *t* test, *n* = 5). (**C**) Corresponding staining of SNP (gray), Dach1 (green) (75), and nuclei (red) in sections of 11-week-old *Ercc1* ctrl and pko kidneys, scale bar indicating 10 μm (*n* = 5). (**D**) Representative immunofluorescence staining of SNP (gray), DNA damage marker gH2A.X (green), and Draq5 (red) in sections of 9-week-old *Ercc1* ctrl and pko kidneys, with quantification of gH2A.X foci per podocyte nucleus and nuclear area, scale bar indicating 2 μm, yellow dotted line indicating nuclear border (unpaired *t* test, *n* = 5, 10 glomeruli per sample, 5 podocytes per glomerulus). (**E**) Representative immunofluorescence staining of SNP (gray), gH2A.X (green), and Draq5 (red) in sections of 11-week-old *Ercc1* ctrl and pko kidneys, with quantification of gH2A.X foci per podocyte nucleus and nuclear area, scale bar indicating 2 μm (unpaired *t* test, *n* = 5, 10 glomeruli per sample, 5 podocytes per glomerulus). (**F**) Representative immunofluorescence staining of nephrin (yellow) with DAPI (gray) of *Ercc1* ctrl at 13 weeks of age and pko kidneys at 7, 9, 11, and 13 weeks of age, scale bar indicating 10 μm (*n* = 5). All violin plots indicate median (black) and upper and lower quartile (gray). *P ≤ 0.05, **P ≤ 0.01, ****P ≤ 0.0001.

Ultrastructural alterations in 9-week-old constitutive *Ercc1*-pko glomeruli were detectable in the form of focally effaced podocyte foot processes. These changes had not yet occurred in 7-week-old animals (Figure 2A). Podocyte number, glomerular volume, and podocyte density, investigated through staining of the podocyte-specific proteins synaptopodin (SNP) and dachshund family transcription factor 1 (Dach1), remained within normal ranges at week 9 (Figure 2B and Supplemental Figure 3A). In contrast, glomeruli from 11-week-old *Ercc1*-pko mice clearly showed severe injury and loss of podocytes, indicated by the decrease and loss of both markers (Figure 2C). This loss of podocytes was further validated through the analysis of WT1-positive cells in 9- and 11-week-old *Ercc1*-pko mice (Supplemental Figure 3B).

Foci of phosphorylated histone 2A.X (gH2A.X), a bona fide marker for DNA double-strand breaks, were significantly increased in both number and area in podocyte nuclei of *Ercc1*-pko glomeruli at week 9 (Figure 2D and Supplemental Figure 3, C–E) compared with control animals. Strikingly, we also observed a smaller number of gH2A.X foci in almost all WT podocyte nuclei, indicative of constant DNA damage occurrence and subsequent repair in healthy glomeruli. At later time points, single podocytes with gH2A.X signals covering larger areas of the nuclei became apparent in *Ercc1*-pko glomeruli (Figure 2E). Further evidence of podocyte stress was revealed by gradual reduction of nephrin abundance, an important protein of the glomerular filtration barrier, from weeks 9 to 13 (Figure 2F and Supplemental Figure 3F).

### DNA damage accumulation in podocytes leads to a shift toward smaller transcripts and activates the mTORC1 pathway.

The cyclic GMP-AMP synthase–stimulator of interferon genes (cGAS/STING) pathway has been shown to play a role in glomerular pathology and can be activated through DNA damage (41). We, hence, investigated whether increased levels of DNA damage in 9-week-old *Ercc1*-pko mice leads to STING phosphorylation (Supplemental Figure 3G). Surprisingly, we did not detect increased levels of phosphorylated STING (p-STING) at both 9 and 11 weeks of age, yet, as an ancillary finding, STING phosphorylation was present in tubular cells of albuminuric 11-week-old *Ercc1*-pko mice.

**Figure 3:**
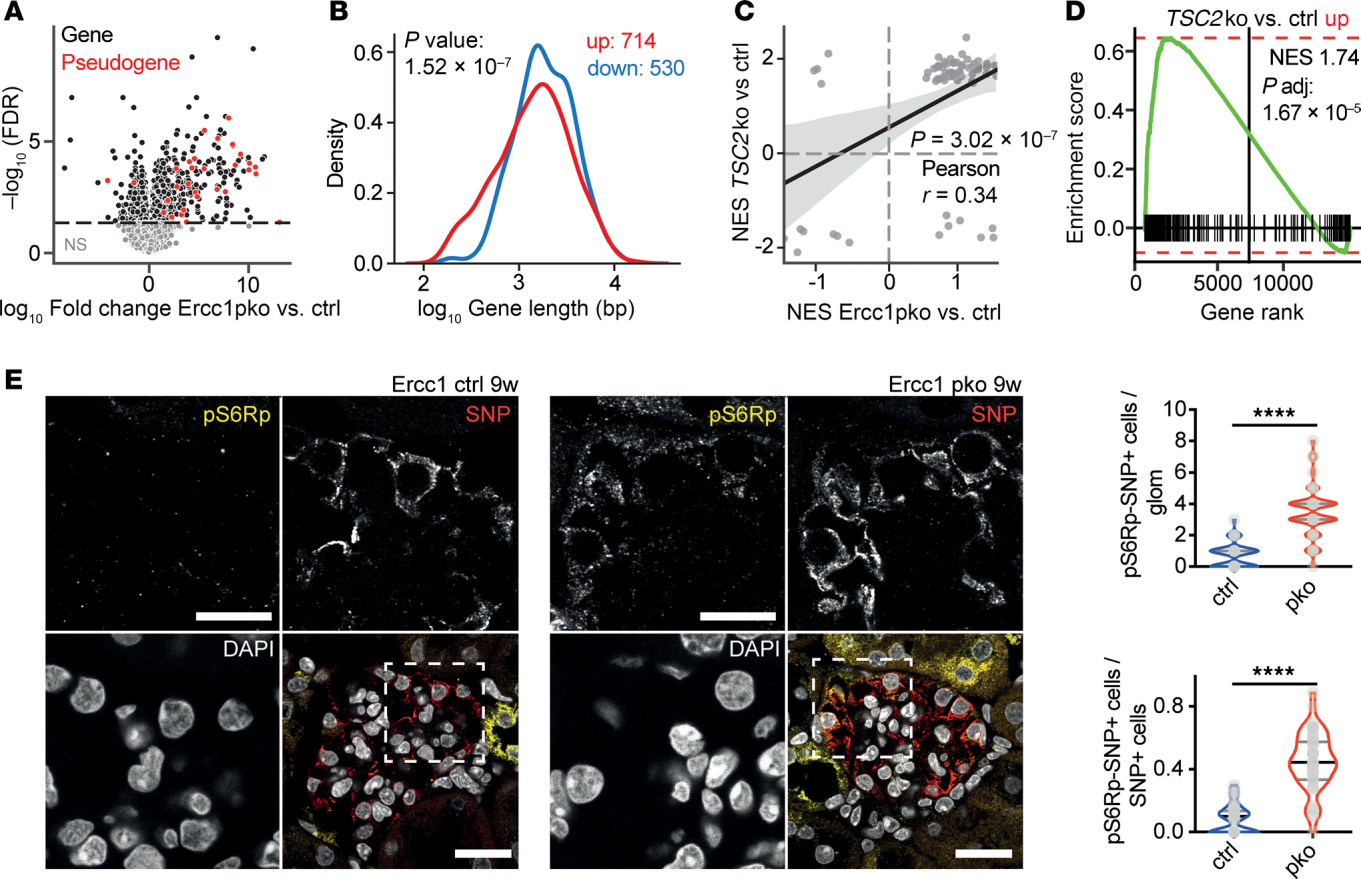
DNA damage accumulation in podocytes leads to a shift toward smaller transcripts and activates the mTORC1 pathway. (**A**) Volcano plot depicting differentially expressed genes of *Ercc1* pko versus control glomeruli. Significantly differentially expressed genes depicted in black, pseudogenes depicted in red. FDR, false discovery rate. (**B**) Gene length analysis of genes differentially expressed in *Ercc1* pko versus control glomeruli. Downregulated genes (blue) are significantly larger than upregulated genes (red). *P* value: 1.52 × 10^–7^ (Mann-Whitney *U* test). (**C**) Correlation analysis plotting the NES of Kyoto Encyclopedia of Genes and Genomes pathway genes in *Tsc2*ko versus ctrl kidneys and *Ercc1* pko versus ctrl glomeruli. (**D**) GSEA of differentially expressed genes (upregulation, fold-change ≥ 2) in *Ercc1*-pko glomeruli classified by the gene rank in the mTORC1 hyperactivation (*Tsc2*ko) data set. The running enrichment score is shown as a green line. The upregulated genes in *Ercc1*-pko glomeruli are significantly (adjusted *P* value: 1.67 × 10^–5^) more enriched (NES: 1.74) in the genes upregulated upon mTORC1 hyperactivation. (**E**) Representative immunofluorescence staining of SNP, p-S6RP, and DAPI in sections of 9-week-old *Ercc1* ctrl and pko kidneys with quantification of SNP and p-S6RP double-positive cells per glomerulus and per total SNP-positive cells, scale bar indicating 20 μm & 10 μm in zoom (unpaired *t* test, *n* = 5, 10 glomeruli per sample). ****P ≤ 0.0001.

To further determine the consequences of DNA damage accumulation on podocyte biology, we performed RNA-Seq analysis on glomeruli isolated from 9-week-old *Ercc1*-pko and ctrl mice. We detected marked changes in gene expression between the 2 groups, with *Ercc1*-pko glomeruli exhibiting an increased expression of pseudogenes, indicative of transcriptional stress and polymerase read-through associated with DNA damage (Figure 3A). When investigating the expression level of transcripts of different lengths, we again detected a shift toward the upregulation of smaller transcripts and the downregulation of longer transcripts, mirroring our findings from *Ercc1*^–/Δ^ glomeruli (Figure 3B). Despite the fact that we recognized varying degrees of nephrin expression at 9 weeks of age, *Ercc1*-pko glomeruli depicted no differences in nephrotic syndrome type 1 (*Nphs1*) expression (data not shown), pointing toward a posttranslational mechanism leading to nephrin protein reduction.

We correlated the acquired data set with publicly available RNA-Seq analyses and recognized a substantial overlap with the gene expression profile obtained from murine *Tsc2*-knockout kidneys (42) both on the level of differentially expressed genes (Supplemental Figure 3H) as well as upregulated pathways (Figure 3C). The *Tsc2* knockout led to a constitutive hyperactivation of the mTORC1 pathway, pointing toward increased mTOR signaling in DNA-damaged podocytes. To further corroborate this, we used the *Tsc2*-knockout data set to establish an mTORC1 hyperactivation gene expression signature and employed this for a GSEA in our dataset. Indeed, the majority of significantly upregulated genes in *Ercc1*-pko glomeruli were enriched within the mTORC1 hyperactivation gene set (Figure 3D).

Podocyte damage and loss have been shown to be tightly linked to mTORC1 activation and cellular hypertrophy of the remaining podocytes (10, 24, 43). With the gene expression profile of 9-week-old *Ercc1*-pko glomeruli indicative of mTORC1 activation, we investigated the phosphorylation of S6 ribosomal protein (S6RP), a downstream target in the mTORC1 pathway, in *Ercc1*-pko mice. Interestingly, we detected a significant increase in p-S6RP^+^ cells in *Ercc1*-pko glomeruli at 9 weeks of age (Figure 3E), a time when no evidence of podocyte loss was present yet (Figure 2C and Supplemental Figure 3B). In-depth analysis revealed that more than 40% of podocytes showed mTORC1 activation at week 9 (Figure 3E).

### mTORC1 activation in podocytes upon genomic stress is mediated through DNA damage signaling kinases DNA-dependent protein kinase and ataxia telangiectasia mutated serine/threonine kinase.

**Figure 4:**
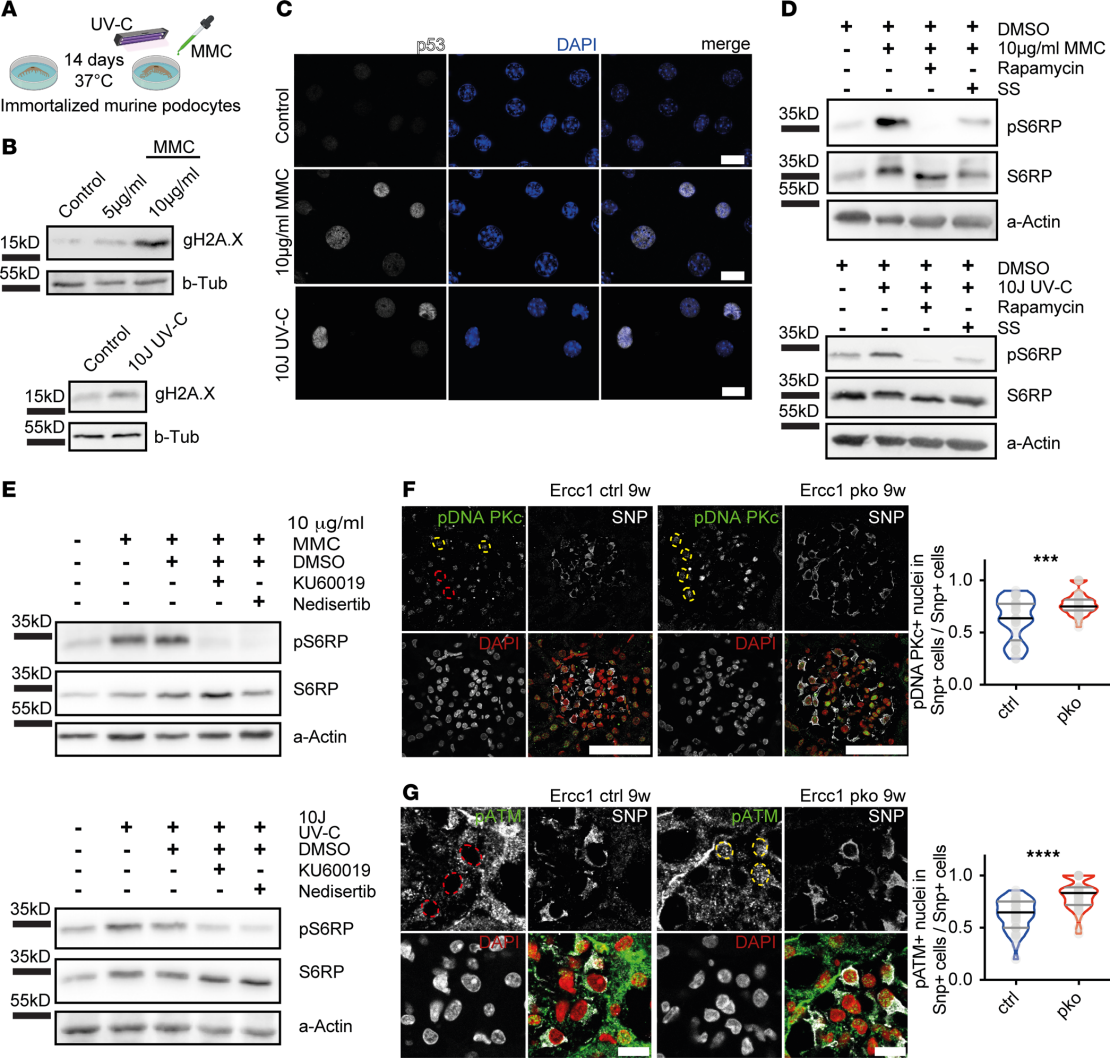
mTORC1 activation in podocytes upon genomic stress is mediated through DNA damage signaling kinases DNA-PK and ATM. (**A**) Schematic in vitro protocol for the induction of DNA damage. (**B**) Representative immunoblot images of gH2A.X and loading control β-tubulin of immortalized murine podocyte lysates (*n* = 3). (**C**) Representative immunofluorescence images for p53 and DAPI in immortalized murine podocytes, scale bar indicating 20 μm (*n* = 3). (**D**) Representative immunoblot images of p-S6RP, S6RP, and loading control protein α-actin of immortalized murine podocyte lysates; p-S6RP and S6RP gels run in parallel (*n* = 3). All cells imaged or lysed after treatment with MMC or UV-C irradiation ± 10 ng/mL rapamycin or serum starvation (SS) (*n* ≥ 4). (**E**) Representative immunoblot images of p-S6RP, S6RP, and loading control protein α-actin of immortalized murine podocyte lysates (*n* = 3 MMC; *n* = 6 UV-C). All cells imaged or lysed after treatment with MMC or UV-C irradiation ± ATM inhibitor KU60019 or DNA-PK inhibitor nedisertib. (**F**) Representative immunofluorescence staining of synaptopodin (gray), p–DNA PKc (green), and DAPI (red) in sections of 9-week-old *Ercc1* ctrl and pko kidneys, with quantification of SNP-positive cells with p–DNA PKc–positive nuclei per total SNP-positive cells. Yellow circles indicating positive nuclei, red circles indicating negative nuclei, scale bar indicating 50 μm (unpaired *t* test, *n* = 4–5, 10 glomeruli per sample). (**G**) Representative immunofluorescence staining of SNP (gray), p-ATM (green), and DAPI (red) in sections of 9-week-old *Ercc1* ctrl and pko kidneys, with quantification of SNP-positive cells with p–ATM-positive nuclei per total SNP-positive cells. Yellow circles indicating positive nuclei, red circles indicating negative nuclei, scale bar indicating 10 μm (unpaired *t* test, *n* = 4–5, 10 glomeruli per sample). All violin plots indicate median (black) and upper and lower quartile (gray). ***P ≤ 0.001, ****P ≤ 0.0001. DNA-PK, DNA-dependent protein kinase; ATM, ataxia telangiectasia mutated serine/threonine kinase.

To further investigate the mechanism behind mTORC1 activation occurring in podocytes upon genomic stress, we induced DNA damage through mitomycin C (MMC) treatment or ultraviolet C (UV-C) irradiation in vitro in immortalized mouse podocytes (Figure 4A). These treatments led to marked increases of gH2A.X (Figure 4B) and accumulation of the DDR protein p53 in the nucleus (Figure 4C). Again, DNA damage induced an increased phosphorylation of S6RP, which is a downstream target of mTORC1, in podocytes (Figure 4D). This phosphorylation was completely abrogated by mTORC1 inhibitor rapamycin and reduced by serum starvation, a well-known mTORC1 modulator (44).

We detected increased autophagy activity upon UV-C–induced DNA damage and decreased activity upon MMC treatment despite mTORC1 activation in both conditions (data not shown), indicative of partially mTORC1-independent regulation of autophagy under genomic stress in vitro.

To identify the link between DNA damage accumulation and mTORC1 activation, we treated MMC- or UV-C–stimulated murine podocytes with inhibitors of the DNA damage signaling cascade (Figure 4E). In both conditions, inhibition of DNA-PK by nedisertib resulted in abrogation of S6RP phosphorylation. Similar results could be achieved with the ATM inhibitor KU60019. No effects were detected upon treatment with the ATM- and Rad3-related (ATR) inhibitor VE822 or with the checkpoint kinase 1 inhibitor prexasertib (data not shown).

To determine the in vivo importance of DNA damage signaling through DNA-PK and ATM, we investigated the activation of both proteins through phosphorylation in immunofluorescence of 9-week-old *Ercc1*-pko mouse kidneys and controls. In line with our in vitro findings, we detected increased staining for both p–DNA PKcs (subunit of the holoenzyme DNA-PK) and p-ATM in podocyte nuclei of *Ercc1*-pko mice (Figure 4, F and G).

These data indicate a role for DNA PKcs and ATM signaling upon DNA damage and a mechanistic link between genomic/transcriptional stress and mTORC1 signaling in podocytes.

### mTORC1 inhibition can decrease DNA double-strand breaks and modulate podocyte damage upon genomic stress.

**Figure 5:**
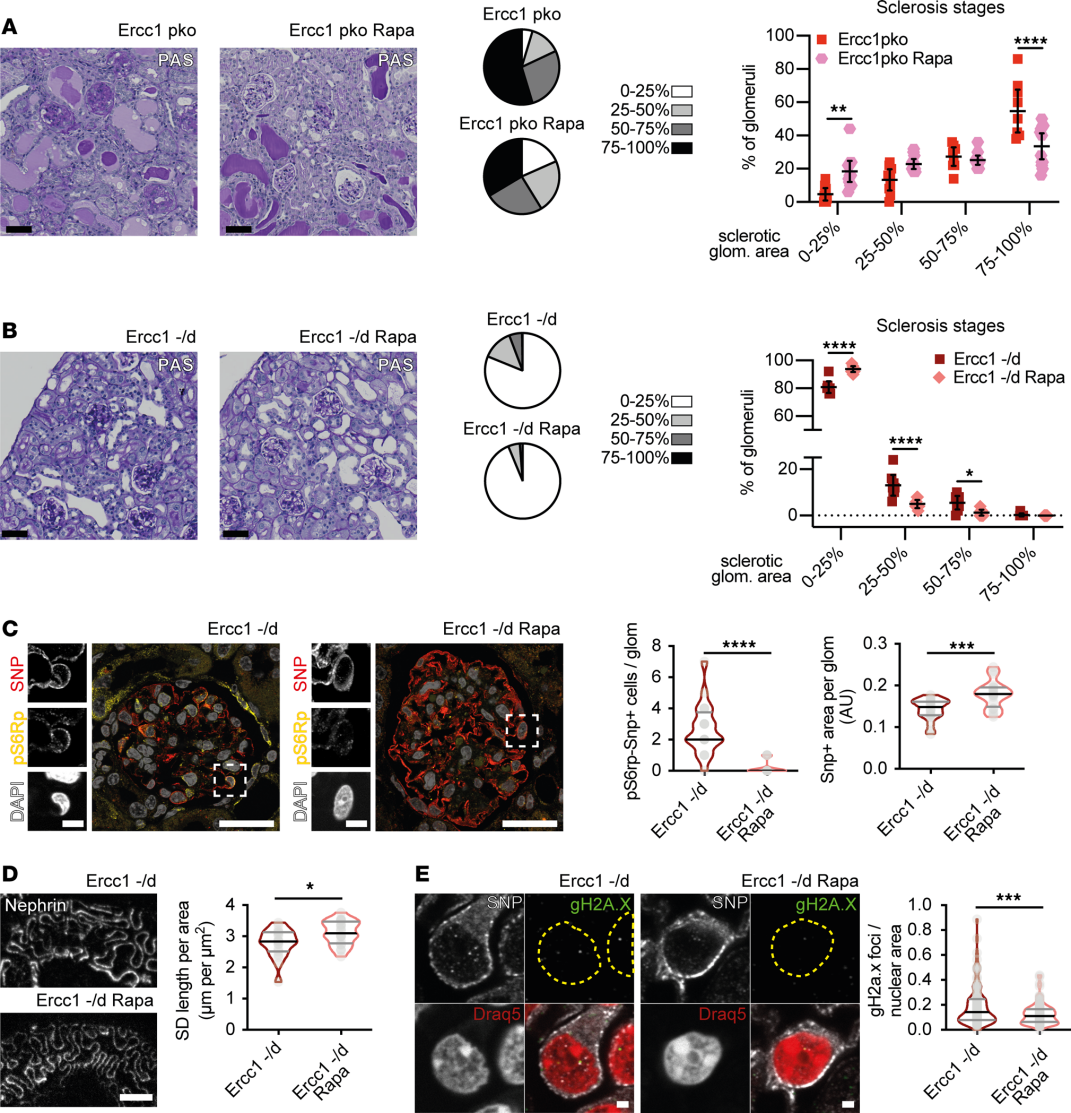
mTORC1 inhibition upon genomic stress can modulate podocyte damage and decrease DNA double-strand breaks. (**A**) Representative PAS staining of *Ercc1*-pko mice treated with vehicle (*Ercc1* pko) or 2 mg rapamycin/kg body weight (*Ercc1* pko Rapa) from 6 weeks of age and glomerulosclerosis assessment of all glomeruli depicted as parts of a whole and scatterplot, scale bar: 50 μm (2-way ANOVA with Šídák’s multiple comparisons test, *n* ≥ 9, 50 glomeruli per sample). (**B**) Representative PAS staining of end-of-life *Ercc1*^–/Δ^ mice treated with 14 mg rapamycin/kg food from 8 weeks of age and glomerulosclerosis assessment of all glomeruli depicted as parts of a whole and scatterplot, scale bar: 50 μm (2-way ANOVA with Šídák’s multiple comparisons test, *n* = 8, 50 glomeruli per sample). (**C**) Representative immunofluorescence staining of SNP, p-S6RP, and DAPI in sections of end-of-life *Ercc1*^–/Δ^ kidneys treated (according to **B**) with quantification of SNP and p-S6RP double-positive cells per glomerulus and SNP-positive area per glomerulus, scale bar: 20 μm, 5 μm in zoom (unpaired *t* test, *n* = 5, 10 glomeruli per sample). (**D**) STED images of cleared kidney tissue of *Ercc1*^–/Δ^ kidneys treated (according to **B**) after immunolabeling with an anti-nephrin antibody with quantification of slit diaphragm length, scale bar: 2 μm (unpaired *t* test, 5 areas per animal, *n* = 4 animals). (**E**) Representative immunofluorescence staining of SNP (gray), gH2A.X (green), and Draq5 (red) in sections of end-of-life *Ercc1*^–/Δ^ kidneys treated (according to **B**) with quantification of gH2A.X foci per nuclear area. Yellow dotted line, nuclear border (*n* = 5, 10 glomeruli per sample, 5 podocytes per glomerulus); scale bar: 2 μm. All violin plots indicate median (black) and upper and lower quartile (gray); scatterplots indicate mean plus 95% confidence interval. *P ≤ 0.05, **P ≤ 0.01, ***P ≤ 0.001, ****P ≤ 0.0001.

To investigate whether increased mTORC1 signaling contributes to the development of podocyte loss, we treated our *Ercc1*-pko mouse model with 2 mg/kg body weight rapamycin via intraperitoneal injections 3 times a week, beginning at 6 weeks of age. We detected a modulation of the glomerular phenotype with a significant reduction in globally sclerotic glomeruli and a shift toward mildly affected glomeruli upon rapamycin treatment at the end of our observation period at week 13 (Figure 5A). Under these rapamycin treatment conditions, albuminuria, serum creatinine, and serum urea levels were not reduced (Supplemental Figure 4, A–C). However, rapamycin therapy was accompanied by a reduction of body weight to normal levels in treated animals (Supplemental Figure 4, D and E), representative of a decreased rate of edema (Supplemental Figure 4F).

In addition, we analyzed archival kidney tissue of *Ercc1*^–/Δ^ mice treated with 14 mg/kg food of the mTORC1 inhibitor rapamycin from 8 weeks of age until termination of the experiment because of high moribund scoring (45). Despite the fact that the treated cohort did not have an extended lifespan, moribund animals of the end-of-life cohort (aged 15–26 weeks), again, presented with a significant reduction of sclerotic glomeruli when treated with rapamycin (Figure 5B). mTORC1 target S6RP phosphorylation was almost absent in podocytes of rapamycin-treated mice (Figure 5C), verifying efficient pathway inhibition, while mTORC2 target PKCα phosphorylation remained unaltered and predominantly present in the mesangial compartment of glomeruli (Supplemental Figure 4G). Functional data through blood and urine were not available for this cohort, so we assessed the effect of mTORC1 inhibition on podocytes during genomic stress by 2 additional methods. First, we analyzed the coverage of podocyte marker protein SNP in glomeruli of untreated versus rapamycin-treated animals and detected increased coverage, a sign of improved podocyte health, in rapamycin-treated glomeruli (Figure 5C). Second, we performed STED (STimulated Emission Depletion; Leica) analysis of the slit diaphragm (SD) and uncovered increased SD length, another indicator of podocyte health, in rapamycin-treated mice (Figure 5D and Supplemental Figure 4H).

**Figure 6:**
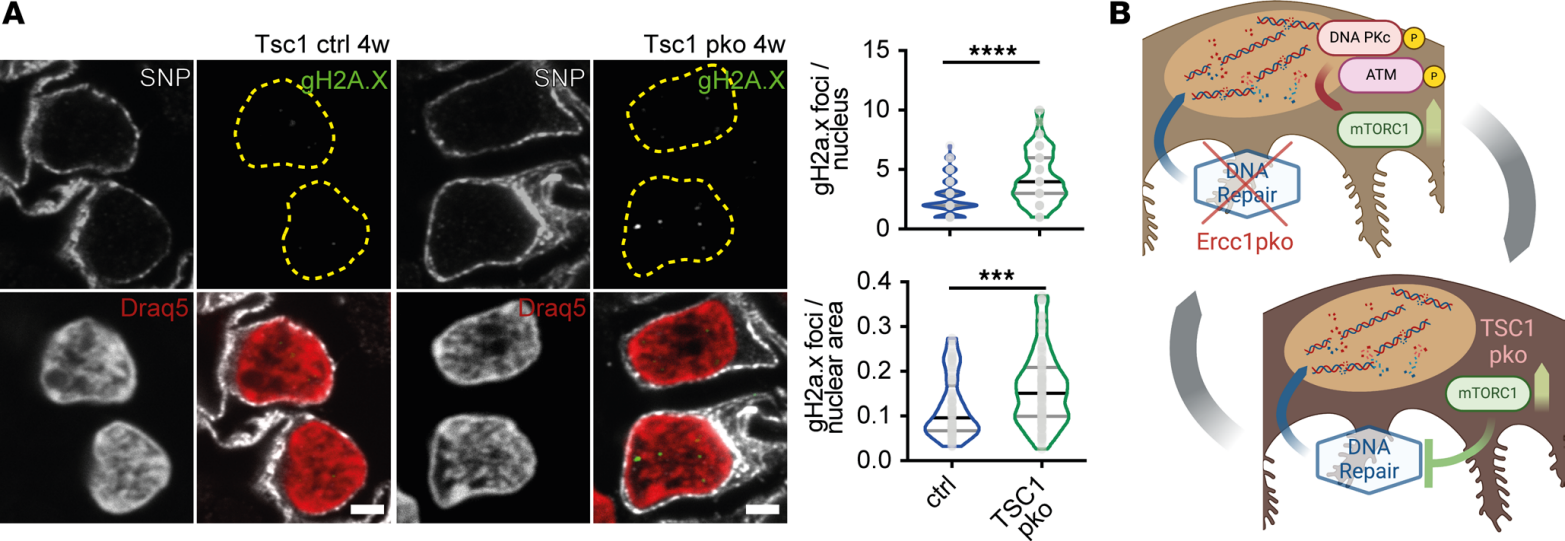
mTORC1 overactivation is associated with increased DNA damage accumulation in podocytes. (**A**) Representative immunofluorescence staining of SNP (gray), DNA damage marker gH2A.X (green), and nuclear marker Draq5 (red) in sections of 4-week-old *Tsc1* ctrl and pko kidneys, with quantification of gH2A.X foci per podocyte nucleus and nuclear area of *Ercc1* ctrl and pko kidneys, yellow dotted line indicating nuclear border (*n* = 5, 10 glomeruli per sample, 5 podocytes per glomerulus), scale bar indicating 2 μm. (**B**) Schematic overview depicting the potential interplay between defective DNA damage repair and increased mTORC1 signaling. In *Ercc1*-pko mice, accumulation of DNA damage triggers DNA damage signaling through DNA PKc/ATM affecting mTORC1 signaling. In *Tsc1*-pko mice, hyperactive mTORC1 signaling associates with increased DNA damage foci. All violin plots indicate median (black) and upper and lower quartile (gray); scatterplots indicate mean plus 95% confidence interval. ***P ≤ 0.001, ****P ≤ 0.0001.

The detection of increased mTORC1 signaling and DNA damage accumulation suggests a potential interplay. It has been shown that increased mTORC1 signaling decreases the cellular ability to repair DNA damage (21, 22). Thus, we investigated to what extent mTORC1 inhibition influences podocyte-specific DNA damage accumulation. Indeed, *Ercc1*^–/Δ^ mice treated with rapamycin exhibited decreased gH2A.X foci in correlation to the observed improved podocyte health (Figure 5E and Supplemental Figure 4I). We assessed the occurrence of increased DNA damage in a podocyte-specific *Tsc1*-knockout (*Tsc1*-pko) mouse model, known to exhibit mTORC1 hyperactivation, by investigating gH2A.X foci accumulation in archival kidney tissue (10, 46). Indeed, *Tsc1*-pko mice also depicted an increased number of DNA damage foci already at 4 weeks of age, when the phenotype was predominantly driven by mTORC1 hyperactivation (Figure 6A).

These data indicate that increased mTORC1 signaling due to DNA damage accumulation and signaling through DNA-PK and ATM and decreased DNA damage repair upon mTORC1 activation may constitute a downward spiral aggravating podocyte injury (Figure 6B).

### DNA damage repair pathways are impaired in focal segmental glomerulosclerosis, resulting in DNA damage accumulation.

We could verify that the well-documented increase in mTORC1 signaling in aged murine WT podocytes coincided with increased detection of DNA damage foci (Supplemental Figure 5, A and C). Similar results were detected in a series of human healthy kidney tissue when comparing a young (21–49 years; *n* = 4) with an aged group (69–81 years; *n* = 5) (Supplemental Figure 5, B and D). Likewise, we detected increased DNA damage signaling through p–DNA-PKcs (Supplemental Figure 5E) and p-ATM (Supplemental Figure 5F) in aged murine podocytes. These results on the level of individual DNA double-strand breaks validated the occurrence of DNA damage in both healthy murine and human podocytes and indicated an association between DNA damage and podocyte aging, presumably contributing to age-related podocyte loss (10). However, we detected fewer p-S6RP^+^ and p-ATM^+^ podocytes in aged mice compared with our *Ercc1*-pko mice. We hypothesize this to result from the retained ability of successful DNA damage repair through Ercc1 in aged WT mice, contributing to the milder phenotype without pronounced glomerulosclerosis compared with the *Ercc1*-pko mice.

**Figure 7:**
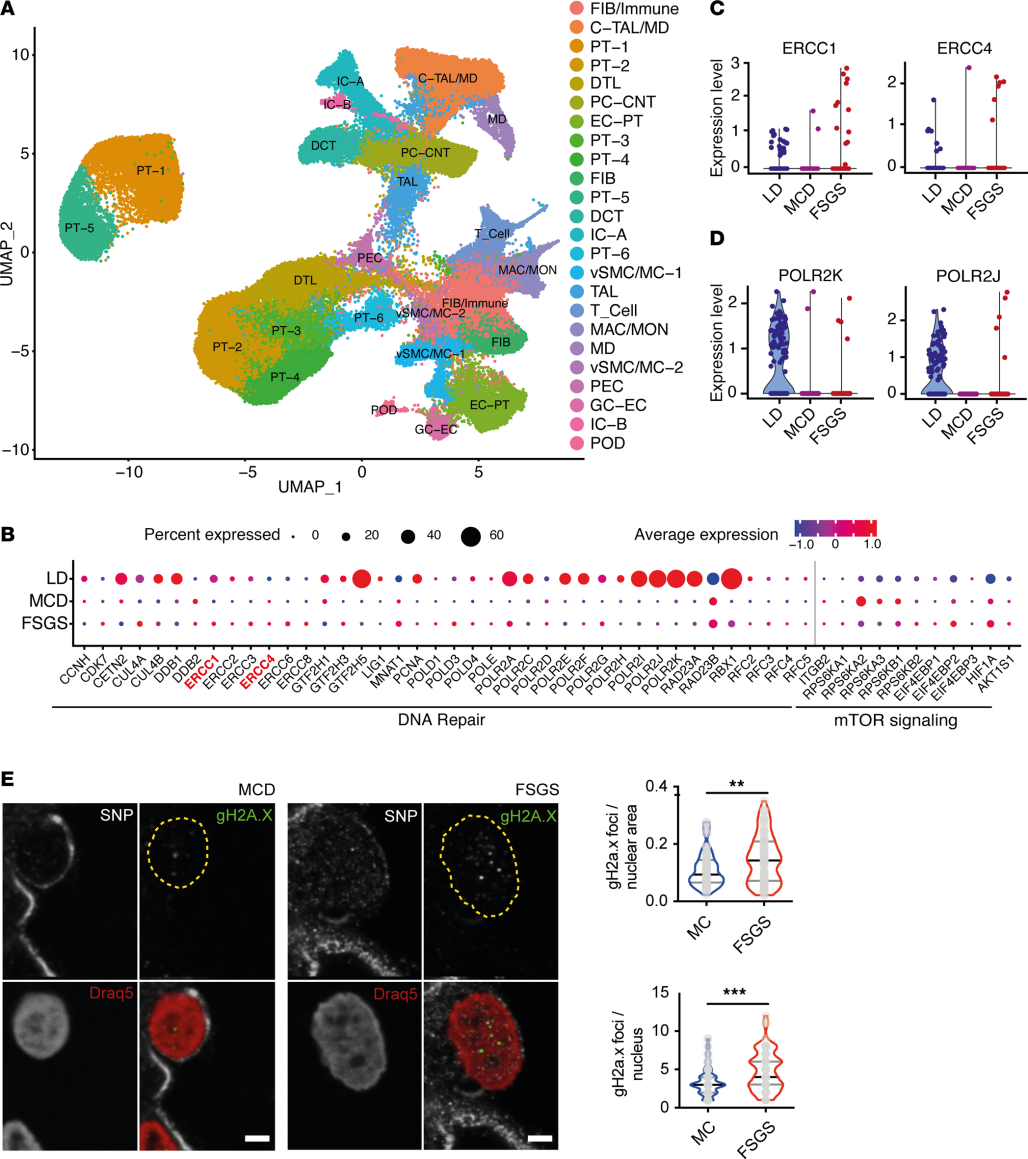
Podocytes accumulate DNA damage during human FSGS. (**A**) Uniform manifold approximation and projection (UMAP) of a single-nucleus sequencing data set (47) depicting 24 cell types in analyzed control, MCD, and FSGS biopsies. FIB/Immune, fibroblasts/immune cells; C-TAL/MD, thick ascending limb of loop of Henle; PT-1–6, proximal tubular cells; DTL, descending thin limb; PC-CNT, connecting tubule; EC-PT, endothelial cell; FIB, fibroblast; DCT, distal convoluted tubule; IC-A, intercalated cell A; vSMC/MC-1, vascular smooth muscle cell/muscle cell 1; TAL, thick ascending limb of loop of Henle; T_Cell, T cells; MAC/MON, macrophage/monocyte; MD, medullary cell; vSMC/MC-2, vascular smooth muscle cell/muscle cell 2; PEC, parietal epithelial cell; GC-EC, glomerular endothelial cell; IC-B, intercalated cell B; POD, podocyte. (**B**) Bubble plot indicating the differences in DNA repair and mTORC1 target gene expression in podocytes between living donor (LD) kidney samples and MCD and FSGS biopsies obtained through single-nucleus sequencing (47). Bubble size indicates percentage of podocytes expressing the target gene, bubble color indicates expression level, and gray line indicates the split between DNA repair and mTORC1 target genes. (**C**) Selected scatterplots of ERCC1 and ERCC4 expression data of single podocytes obtained from LD, MCD, and FSGS biopsies. (**D**) Selected scatterplots of RNA polymerase 1–3 subunit POLR2K and RNA polymerase 2 subunit POLR2J of single podocytes obtained from LD, MCD, and FSGS biopsies. (**E**) Representative immunofluorescence staining of SNP, gH2A.X, and Draq5 in sections of human MCD and FSGS biopsies, yellow dotted line indicating nuclear border, scale bar indicating 2 μm with quantification of gH2A.X foci per podocyte nuclear area and per podocyte nucleus in human MCD and FSGS biopsies (unpaired *t* test, *n* = 4, 4 glomeruli per sample, 5 podocytes per glomerulus). All violin plots indicate median (black) and upper and lower quartile (gray). **P ≤ 0.01. ***P ≤ 0.001.

The striking podocyte phenotype upon DNA damage accumulation, nevertheless, prompted us to investigate DNA damage repair genes in different glomerular diseases. When analyzing data of a human renal cDNA biobank (European Renal cDNA Bank, ERCB), which contains cDNA samples of kidney biopsies, we detected alterations in the expression of DNA repair genes in glomerular lysates of different nephropathies compared with controls (Supplemental Figure 6 and Supplemental Table 1), with the strongest effect in focal segmental glomerulosclerosis (FSGS) and the mildest in minimal change disease (MCD). Hence, we investigated the expression of DNA repair genes in podocytes of human FSGS biopsies, MCD, and healthy controls of a single-nucleus RNA-Seq data set (47) (Figure 7A). Indeed, we detected an upregulation in *Ercc1–8* genes, all involved in damage recognition, DNA unwinding, and damage excision (Figure 7, B and C, and Supplemental Figure 7), in FSGS podocytes. This upregulation indicates that podocytes increase their machinery to repair DNA damage in the presence of FSGS. Vice versa, the expression of several subunits of RNA polymerase 2 and the shared subunit of RNA polymerases 1–3, *POL2RK*, was virtually lost in human podocytes of both MCD and FSGS samples (Figure 7, B and D, and Supplemental Figure 7). These expression data indicate a predominance of damage recognition and strand excision in FSGS biopsies with the potential of downregulation/stalling of RNA synthesis. Targets of the mTORC1 pathway served as an internal control, depicting increased expression with little change in the percentage of podocytes expressing these genes (Figure 7B).

To further elucidate this observation of perturbed DNA damage recognition, we investigated the accumulation of DNA damage in podocyte nuclei of human biopsies of patients with FSGS compared with MCD using gH2A.X immunofluorescence staining. Indeed, we detected a marked increase in podocyte-specific nuclear gH2A.X foci in FSGS glomeruli (Figure 7E), corresponding to increased DNA double-strand breaks and thus suggestive of an involvement of DNA damage accumulation in human glomerular disease with marked podocyte damage and loss.

In line with this, using an expression quantitative trait locus (eQTL) analysis, we identified single nucleotide polymorphisms associated with alterations in the expression of DNA repair genes (Supplemental Tables 2–4) in sclerotic glomerular diseases that could be targets of interest for future investigations and for therapeutic modulation.

## Discussion

To which extent glomerular epithelial cells are subject to transcription stress is currently unclear. Elucidating this subject is of particular interest for podocytes, terminally differentiated cells with a limited ability to self-renew (7). These cells have to maintain a functional genome for the organism’s entire lifespan, which is specifically important in the later stages of life. Our study reveals the occurrence of individual DNA damage foci in podocyte nuclei under healthy conditions, indicating a need for constant DNA maintenance and repair and an increase of these DNA damage foci in aged podocytes. Furthermore, we show that this accumulation of DNA damage results in the newly established principle of transcriptional stalling and a shift to the expression of shorter transcripts in glomeruli (35). Recent studies have indicated that the aged podocyte phenotype is in part characterized by senescence. Transcriptomic analysis detected differential expression of senescence-associated genes in aged podocytes (48) coinciding with increased gene silencing. This senescence phenotype was shown to be driven by GSK3β overexpression, and inhibition via lithium could improve podocyte aging in mice and decrease podocyturia in humans (49). This fits well with the interplay of DNA damage and GSK3β activation, as GSK3β was shown to be involved in DNA damage repair in cancer, and its nuclear translocation was, in part, dependent on p53 activation and nuclear translocation (50–52). A potential causal chain for the senescent phenotype could, therefore, be the activation of GSK3β signaling due to DNA damage accumulation in aged podocytes.

The response to transcriptomic stress in podocytes was different from the pattern reported in other cell types, as podocytes activated mTORC1 signaling upon DNA damage. The interplay of DNA damage, its repair, and the mTOR pathway has been the subject of numerous studies, specifically in the field of cancer biology. These studies indicated that mTORC1 signaling is inhibited upon DNA damage in a TSC-, Sestrin-, or AKT-dependent manner (18–20). Strikingly, we observed that podocytes both in vitro and in vivo reacted to endogenous accumulation or exogenous infliction of DNA damage with activation of the mTORC1 pathway. Herein lies a fundamental difference from past reports and a potential disease mechanism, as numerous studies have depicted the importance of a tight regulation of mTORC1 for podocyte health and the deleterious effects of both overactivation and repression in disease (10, 24, 26, 43, 53, 54). Since mTORC1 activation occurs in *Ercc1*-pko podocytes at 9 weeks, a time point of no overt podocyte loss, mild ultrastructural differences, and significant accumulation of DNA damage foci, our data indicate a link between transcription stress and mTORC1 activation in podocytes. This pathway upregulation takes place through the activation of DNA-PK, a nuclear serine/threonine protein kinase, and ATM in vitro, with both kinases also being activated in vivo.

A growing body of evidence suggests that mTORC1 activity reduces the capacity of successful DNA repair (21, 22), e.g., through ribosomal S6 kinase–dependent phosphorylation of E3 ubiquitin-protein ligase RNF168 (23). However, upon podocyte depletion, remaining podocytes on the glomerular tuft counteract the loss of neighboring cells through mTORC1-mediated hypertrophy (10), which could impair proper DNA repair. In line with this, *Ercc1*^–/Δ^ mice showed significantly lower levels of DNA damage foci under mTORC1 inhibition (Figure 5E). This is further underlined by *Tsc1*-pko animals displaying mTORC1 hyperactivation. These animals accumulate a significant amount of DNA damage foci already at 4 weeks of age (Figure 6A). We, therefore, hypothesize that the interplay of DNA damage and mTORC1 signaling can lead to a downward spiral: Accumulation of DNA damage in podocytes triggers mTORC1 activation, which further aggravates insufficient DNA maintenance, leading to excessive podocyte loss. This cascade could be modulated through well-timed mTORC1 inhibition as suggested by the ameliorated SNP expression, increased SD length, and reduction of glomerulosclerosis in rapamycin-treated *Ercc1*-deficient animals. Another way to halt or reverse the proposed downward spiral could be the upregulation of DNA repair mechanisms to alleviate accumulated transcription stress or the timed inhibition of DNA damage signaling through DNA-PK or ATM inhibition.

Only recently has the importance of DNA repair sparked great interest in the field of podocyte biology. The description of glomerular abnormalities in human syndromes caused by mutations in DNA repair genes is scarce because of their frequently lethal phenotypes (55, 56). The first evidence of ERCC1 being an essential protein in kidney health was provided through the identification of *ERCC1* variants causing proximal tubular dysfunction. Besides that, the patients investigated had glomerular disease (57). Data from whole-body *Ercc1*-null mice with correction of the liver phenotype also indicate a role for glomerular health, with *Ercc1*-deficient animals rapidly dying of renal failure (58). Beyond that, there is evidence for podocyte involvement in human syndromes caused by mutations of DNA repair genes with patients exhibiting proteinuria and nephrotic syndrome (37, 59, 60). Consistently, we could report that gene deletion of DNA repair endonuclease cofactor *Ercc1* in podocytes caused proteinuria and FSGS in our mouse model though a preprint of this manuscript (61), as confirmed by Hama et al. (62).

The same holds true for factors involved in other forms of DNA repair, such as the KEOPS complex (14, 63), *KAT5* (64), and *SMPDL3b* in podocytes, which all play a role in DNA damage recognition in DNA damage recognition (65). Recently, epigenetic regulation was also shown to be involved in genome maintenance, as *HDAC* deletion in podocytes resulted in DNA damage accumulation, senescence, cell cycle reentry, and FSGS (66). Beyond that, podocyte specific DNA damage was shown to modulate the renal immune microenvironment (67). Itoh and coworkers linked proteinuric kidney disease present in patients with hypertension and diabetes to DNA double-strand breaks and methylation in the promotor region of the SD protein nephrin, and they established an association to DNA double-strand breaks in glomeruli of patients with IgA nephropathy (15, 16). Our analysis adds considerably to this body of evidence, as we identified multiple factors of DNA maintenance to be transcriptionally altered in glomeruli of different glomerular diseases. It is noteworthy that the fewest transcriptional alterations were detected in MCD, a potentially reversible podocyte disorder, while all other glomerular diseases investigated, characterized by podocyte loss, depicted perturbation in 40%–70% of DNA repair genes. Expanding on this analysis to the transcriptional profile of single podocytes in MCD and FSGS, we found increased expression of DNA repair proteins in FSGS, suggesting that increased transcription stress may contribute to the loss of podocytes in renal disease. This is in line with altered expression of endonuclease ERCC4 in IgA nephropathy and a recent report on gH2A.X accumulation in organoid podocytes incubated with primary or recurrent FSGS plasma (15, 68).

Our study complements these data suggesting that loss of especially transcription-coupled nucleotide excision repair capacity, which removes lesions from the template DNA strands of actively transcribed genes, may contribute to the progression of some types of glomerular disease and aging. The importance of DNA damage repair in podocyte homeostasis is consistent with the role in other postmitotic cell types and particularly apparent in neurodegenerative pathologies typical for DNA repair deficiency syndromes (5). Repairing transcription-blocking lesions might thus play a pivotal role in podocytes that need to maintain the integrity of transcribed genes during the entire lifespan of the organism. Even hepatocytes of *Ercc1*^–/Δ^ mice, usually characterized by high self-renewing potential, showed a considerable block of transcription, indicative of transcription-coupled mechanisms being stalled (47, 69). Potential sites in the genome that may be used to alter glomerular damage repair were already identified in our eQTL analysis in patients with FSGS. This is of particular interest since there seems to be a broad interplay between gene products exerting functions beyond their canonical pathways in genome maintenance (70). Likewise, decreased expression of DNA repair genes in a subset of patients or the accumulation of DNA damage through the aging process may lead to increased mTORC1 activation in podocytes, thereby rendering a subgroup of patients vulnerable to the development of glomerular disease and scarring.

In conclusion, we identified efficient DNA damage repair as an essential stress response mechanism for podocyte homeostasis and established a link between DNA damage accumulation and mTORC1 signaling. Furthermore, the presented study identifies transcription stress as a hallmark of podocyte aging, loss, and glomerular disease, suitable for precision medicine approaches.

## Methods

For detailed methods please see Supplemental Methods.

### Sex as a biological variable.

Our study examined male and female animals, and similar findings are reported for both sexes.

### GSEA.

Raw data were downloaded from National Center for Biotechnology Information (NCBI) Gene Expression Omnibus (GEO) GSE43061, normalized with RMA (71), and further processed with limma (72). The log fold-changes of the comparison of *Ercc1* 14 weeks versus WT 14 weeks were used as input for the gene-length GSEA (17).

### Mice.

Mice were bred in a mixed FVB/CD1 (*Ercc1* pko) or FVB/CD1/C57BL/6 (*Ercc1* ipko) background. Aged WT mice were obtained through Janvier Labs, Le Genest-Saint-Isle, France. All offspring were born in normal Mendelian ratios. Spot urine was collected once a week during cage changes or during sacrifice. Tamoxifen was administered at 400 mg/kg tamoxifen in dry chow starting when mice were 8 weeks of age for a total of 4 weeks. Nphs2.Cre (pod:cre) and podocin-iCreER(T2) (ind.pod:cre) mice were provided by Department II of Internal Medicine and Center for Molecular Medicine Cologne, University of Cologne, Faculty of Medicine and University Hospital Cologne, Cologne, Germany (53) . *Ercc1*^fl/fl^ mice were provided by Institute of Molecular Biology and Biotechnology, Foundation for Research and Technology-Hellas, Heraklion, Crete, Greece (38).

For rapamycin injection studies male and female *Ercc1*^fl/fl^ (ctrl) and *Ercc1* *Nphs2.Cre* (pko) mice at 6 weeks of age were injected intraperitoneally 3 times per week with 2 mg/kg body weight of rapamycin diluted in 5% ethanol, 5% Tween 80, and 5% PEG 400 or with 5% ethanol, 5% Tween 80, and 5% PEG 400 as vehicle. Mice were sacrificed at 13 weeks of age for serum and kidney tissue isolation.

Mice were anesthetized by intraperitoneal injection of 10 μL/g body weight of 0.01% xylocaine and 12.5 mg/mL ketamine; blood was drawn from the left ventricle, and animals were perfused with cold phosphate-buffered saline (PBS). Kidneys were excised and embedded in OCT (Sakura) and frozen at –80°C or fixed in 4% neutral buffered formalin and subsequently embedded in paraffin.

For electron microscopy mice were perfused with 4% paraformaldehyde and 2% glutaraldehyde in 0.1 M sodium cacodylate, pH 7.4.

Archival tissue of *Tsc1*-pko mice was provided by the University of Freiburg, Germany. *Tsc1*^fl/fl^ mice were crossbred with Nphs2-Cre^+^. For further details see ref. 46.

Tissue of *Ercc1*^–/Δ^ mice was provided by the National Institute for Public Health and the Environment, the Netherlands. Further details are provided in ref. 45.

### Podocyte isolation.

*Ercc1*^fl/fl^ mice heterozygous for the *R26mTmG* and *Nphs2.Cre* transgene were sacrificed, and kidneys were used for glomerular preparation, as previously described (73). The glomeruli were digested and the single-cell suspension was used for FACS.

### qPCR analysis.

Total RNA was extracted from podocytes using Direct-zol RNA MiniPrep Kit (catalog R2052, Zymo Research). cDNA was synthesized with High Capacity cDNA Reverse Transcription Kit (catalog 4368814, Applied Biosystems). PCR was performed using TaqMan Gene Expression Master Mix (catalog 4369016, Applied Biosystems) and the Applied Biosystems Real-time PCR system.

### Urinary albumin ELISA and creatinine measurement.

Urinary albumin levels were measured with a mouse albumin ELISA kit (mouse albumin ELISA kit; Bethyl Labs). Urinary creatinine kit (Cayman Chemical) was used to determine corresponding urinary creatinine values. For Coomassie blue detection of albuminuria, spot urine of mice was diluted 1:20 in 1× Laemmli buffer.

### Plasma creatinine and urea measurement.

Creatinine and urea were measured using standard clinical protocols by the Department of Clinical Chemistry of the University of Cologne.

### Histologic analysis.

To assess morphological changes in light microscopy we performed PAS staining.

### Immunofluorescence staining.

Paraffin-embedded tissue was cut into 3 μm–thick sections and processed according to published protocols (74). Primary antibodies were used at 1:200 dilution: anti-gH2A.X 2577s Cell Signaling Technology, anti-nephrin GP-N2 Progen, anti-synaptopodin 65294 Progen, anti-Dach1 HPA012672 Sigma-Aldrich (75), anti–phospho-S6 Ribosomal Protein (Ser235/236) 4858 Cell Signaling Technology, anti–phospho-STING (Ser336) 19781 Cell Signaling Technology, anti–phospho-PKCα (Ser657) sc-12356 Santa Cruz Biotechnology, anti-p53 p53-protein-cm5 Leica Biosystems, anti-ATM (phospho S1987) antibody [EPR28058-71] ab315019 Abcam, anti-DNA PKcs (phospho S2056) antibody - ChIP Grade ab18192 Abcam. DAPI or far-red fluorescent DNA dye Draq5 (ab108410 Abcam) was used as a nuclear marker.

Cells were processed according to published protocols (54).

### Glomerular isolation and RNA-Seq analysis.

Glomerular isolation was performed according to the previously described protocol (73) and flash-frozen in liquid nitrogen for storage. Glomeruli were lysed in TRIzol (Invitrogen), and total RNA was extracted using the Direct-zol RNA MiniPrep kit according to manufacturer’s instructions. Libraries were prepared from 500 ng total RNA. For RNA-Seq analysis see Supplemental Methods.

The RNA-Seq data are available from the GEO under the accession number GSE292420.

### In vitro experiments.

Conditional immortalized murine podocytes were a gift by Stuart Shankland (Division of Nephrology, University of Washington, Seattle, Washington, USA). Cells were cultured as previously described (76). After 10 days of differentiation, cells were treated with 5 or 10 μg/mL MMC (M0503 Sigma-Aldrich) for 2 hours in serum-free medium, followed by 1 washing step with PBS and another 6 hours of incubation in serum-free medium without MMC before further processing. For experiments with DDR inhibitors, differentiated cells were pretreated with 3 μM KU60019 (Selleckchem) or 1 μM nedisertib (Selleckchem) for 1 hour before inducing DNA damage by UV-C or MMC treatment. Inhibitors were added again after medium change following DNA damage induction to further incubate cells for 6 hours before cell lysis.

### Western blot analysis.

SDS-PAGE was used for protein size separation with subsequent blotting onto PVDF membranes and visualized with enhanced chemiluminescence after incubation of the blots with corresponding antibodies: Phospho-Histone H2A.X (Ser139); Phospho-S6 Ribosomal Protein (Ser235/236) (D57.2.2E); S6 Ribosomal Protein (5G10) from Cell Signaling Technology; α-actin (JLA20) from Developmental Studies Hybridoma Bank; and β-tubulin (E7) from Developmental Studies Hybridoma Bank.

### Preparation of formalin-fixed, paraffin-embedded tissue and STED microscopy.

Kidney tissue samples were prepared according to a previously published protocol, with slight modifications (77). Samples were incubated in a sheep polyclonal antibody against nephrin (R&D Systems, Bio-Techne, AF4269). Images were acquired using a Leica SP8 3X gSTED system using a 100× 1.4 NA objective. The SD length was measured using an ImageJ (NIH) macro as done previously (8).

### ERCB human microarray analysis.

A total of 167 genes involved in DNA repair and nucleotide excision repair were compiled from the Hallmark gene set “DNA-Repair” from the Molecular Signatures Database (78) and from literature research. Analysis included datasets from patients with MCD (*n* = 14), FSGS (*n* = 23), membranous nephropathy (*n* = 21), IgA nephropathy (*n* = 27), and hypertensive nephropathy (*n* = 15) as well as controls (LD; *n* = 42) (GSE99340, LD data from GSE32591, GSE37463). The resulting gene expression list was censored for genes whose products were detected in a transcriptomic and proteomic analysis of WT murine podocytes (73).

### Single-nucleus sequencing.

Nuclei were prepared from kidney biopsy cores stored in RNAlater from patients with FSGS and MCD enrolled in the Nephrotic Syndrome Study Network (NEPTUNE) study (47). The processing followed the protocol developed from the Kidney Precision Medicine Project (KPMP). A comparison of these cluster-selective gene profiles was made against previously identified cell marker gene sets from human kidney samples from KPMP and other sources (47).

### eQTL analysis.

For the subgroup analysis of FSGS cohort, the procedure described in Gillies et al., 2018 (79), was used with the following exceptions: Only patients with FSGS were analyzed (*n* = 87), and only RNA-Seq expression data for glomerular samples were utilized.

### Study approval.

All investigations involving human specimens have been conducted according to the Declaration of Helsinki following approval of the local ethics committees. Written informed consent was received from participants prior to inclusion in the study. All mouse experiments were conducted according to institutional and federal guidelines and approved by the Landesamt für Natur, Umwelt und Verbraucherschutz Nordrhein-Westfalen Vogelschutzgebiet, Düsseldorf, Germany 84-02.04.2013.A336.

### Statistics.

If not stated otherwise, unpaired 2-tailed *t* test was used to compare 2 groups, and *P* ≤ 0.05 was considered significant. For multiple-group comparisons, we applied 1-way ANOVA or 2-way ANOVA where applicable followed by Tukey’s post hoc correction. Statistics were performed using GraphPad Prism 8–10.

### Data availability.

All expression data are available through GEO under the accession numbers GSE43061, GSE244072, GSE292420, GSE213030.

All additional data displayed in the figures are provided in the file titled “Supporting Data Values.”

## Author contributions

FB, AMM, LB, RAH, AR, RE, DUJ, VGP, DHM, MNW, GC, HC, MR, DF, GGS, SK, MTL, WB, VN, and PJM performed experiments; FB, LB, AMM, GC, RAH, DUJ, VGP, MNW, DF, MTL, WB, MGS, and VN analyzed data; FB, AMM, VGP, GAG, METD, PTB, CDC, M Kann, MGS, M Kretzler, TBH, B Schermer, T Benzing, B Schumacher, and CEK conceived experiments; FB, VGP, B Schumacher, and CEK wrote the manuscript; all authors revised the manuscript; and all authors agreed on the publication in the presented state.

## Supplementary Material

Supplemental data

Unedited blot and gel images

Supplemental table 1

Supplemental table 2

Supplemental table 3

Supplemental table 4

Supporting data values

## Version 1 05/20/2025

In-Press Preview

## Version 2 06/23/2025

Electronic publication
