# Supplementary material for: Loss of genome maintenance is linked to mTOR complex 1 signaling and accelerates podocyte damage
Source: JCI Insight. 2025 May 20;10(12):e172370. doi: 10.1172/jci.insight.172370 (PMC12220965; doi:10.1172/jci.insight.172370)
Supplement: Supplemental data [file jciinsight-10-172370-s066.pdf]

Supplementary Information 172370-INS-RG-RV-4:

Fig S1: Glomerular DNA damage causes a shift towards expression of shorter transcripts

GSEA of gene classes according to transcript length. Shown are the analyses for the shortest 5 % (top left), shortest 1 % (top right), longest 5% (bottom left), and longest 1% (bottom right) of genes. The bottom of each panel shows the log2 fold changes of the microarray data in a ranked order. The top panels show the running enrichment score as an orange line. The smallest 1% (normalized enrichment score (NES) of 2.01) and 5% (NES of 1.37) of genes are significantly more enriched in the upregulated genes, while the longest 1% (NES of -1.69) and 5% (NES of -1.42) of genes are significantly enriched in the downregulated genes in the comparison *Ercc1* -/delta 14 weeks vs. WT 14 weeks.

Figure S2: Podocyte-specific Ercc1 deletion causes kidney failure and glomerulosclerosis

A: Breeding scheme for homozygous and heterozygous podocyte-specific *Ercc1* pko mice.
B: serum creatinine analysis; C: serum urea analysis of *Ercc1* ctrl and pko mice (both two-way ANOVA with Šídák's multiple comparisons test, n=4-9).
D: quantification of sclerotic glomeruli of *Ercc1* ctrl and pko mice (two-way ANOVA with Šídák's multiple comparisons test, n = 5, 50 glomeruli per sample).
E: Representative Coomassie blue staining of *Ercc1* ctrl and wt/pko (het) urine at 36 weeks of age; bovine serum albumin (BSA) was loaded as reference.
F: Representative Periodic Acid Schiff (PAS) staining of *Ercc1* wt/pko (het) kidney at 72 weeks of age, scalebar: 100 μm.
G: Breeding and induction scheme for homozygous inducible podocyte-specific *Ercc1* ko mice (ipko).
**p ≤ 0,01, ***p ≤ 0,001, ****p ≤ 0,0001, scalebar: 100μm, Scatterplots indicate mean plus 95% confidence interval

Figure S3: The podocyte-specific knockout of Ercc1 leads to podocyte loss and mTORC1 activation

A: Quantification of glomerular volume of 9-week-old *Ercc1* ctrl and pko kidneys, (unpaired t-test, n = 5, 10 glomeruli per sample).
B: Quantification of WT+ nuclei per glomerulus of *Ercc1* ctrl and pko kidneys at 9 and 11 weeks of age, (unpaired t-test, n = 4, ≥50 glomeruli per group).
C: Quantification of podocyte nuclear area of 9-week-old *Ercc1* ctrl and pko kidneys, (unpaired t-test, n = 5, 10 glomeruli per sample, 5 podocytes per glomerulus).
D: Quantification of gH2A.X foci area per podocyte nucleus of 9-week-old *Ercc1* ctrl and pko kidneys, (unpaired t-test, n = 5, 10 glomeruli per sample, 5 podocytes per glomerulus).
E: Quantification of gH2A.X foci area per podocyte nuclear area of 9-week-old *Ercc1* ctrl and pko kidneys, (unpaired t-test, n = 5, 10 glomeruli per sample, 5 podocytes per glomerulus).
F: Quantification of nephrin positive area in μm^2 of *Ercc1* ctrl and pko kidneys at 7, 9, 11, and 13 weeks of age, (unpaired t-test, n=4, 10 glomeruli per sample).
G: Representative immunofluorescence staining of SNP, pSTING and DAPI in sections of 9-week-old *Ercc1* ctrl and pko kidneys and 11-week-old *Ercc1* pko kidneys, scalebar indicating 10 μm
H: Correlation analysis plotting the normalized enrichment score (NES) of differentially expressed genes in TSC2ko vs ctrl kidneys and Ercc1pko vs ctrl glomeruli.
All violin plots indicate median (black) and upper and lower quartile (gray), **p ≤ 0,01, ***p ≤ 0,001, ****p ≤ 0,0001.

Figure S4: mTORC1 inhibition upon genomic stress can modulate podocyte damage and ameliorate their morphology

A: urinary albumin/creatinine analysis; B: serum creatinine analysis; C: serum urea analysis and weight analysis (D: female; E: male) of *Ercc1* pko mice treated with vehicle (Ercc1pko) or rapamycin (Ercc1pko Rapa), (A: one way ANOVA Tukey's multiple comparisons test, B-F: unpaired t-test, A-C: n = 8-12, D: n=5-7, E: n=4-6).

F: Representative image of rapamycin and vehicle treated *Ercc1* pko mice at 13 weeks of age depicting edema in the vehicle treated animal.

G: Representative immunofluorescence staining of SNP, pPKC alpha and DAPI in sections of end-of-life *Ercc1* -/delta kidneys treated with 14 mg rapamycin per kg food from 8 weeks of age scalebar: 20μm, (n=5).

H: STED images of cleared kidney tissue of *Ercc1* -/delta kidneys treated with 14 mg rapamycin per kg food from 8 weeks of age after immunolabelling with an anti-nephrin antibody. Zoom depicted in figure 4D indicated by dashed yellow boxes, scalebar: 5μm.

I: Quantification of gH2A.X foci per podocyte nucleus in sections of end-of-life *Ercc1* -/delta kidneys treated with 14 mg rapamycin per kg food from 8 weeks of age, (n=5, 10 glomeruli per sample, 5 podocytes per glomerulus),

All scatterplots indicate mean plus 95% confidence interval, all violin plots indicate median (black) and upper and lower quartile (gray), **p ≤ 0,01, ****p ≤ 0,0001,

Figure S5: Podocytes accumulate DNA damage accompanied by increased DNA PKc, ATM and mTORC1 signaling during aging

A: Representative immunofluorescence staining of SNP, pS6RP and DAPI in sections of 14-week-old versus 96-week-old wildtype (WT) mice kidneys with quantification of SNP and pS6RP double positive cells per glomerulus and per total SNP positive cells, scalebar indicating 50 μm & 30 μm in zoom (unpaired t-test, n=4, 10 glomeruli per sample).

B: Representative immunofluorescence staining of SNP, pS6RP, and DAPI in sections of young and old human tumor nephrectomy kidneys with quantification of SNP and pS6RP double positive cells per glomerulus and per total SNP positive cells, scalebar indicating 10 μm, 5 μm in zoom, (unpaired t-test, n=≥4, 10 glomeruli per sample).

C: Representative immunofluorescence staining of SNP, gH2A.X, and Draq5 in sections of murine young and aged wildtype kidneys with quantification of gH2A.X foci per podocyte nuclear area, scalebar indicating 2 μm, (unpaired t-test, n=4, 5 glomeruli per sample, 5 podocytes per glomerulus).

D: Representative immunofluorescence staining of SNP, gH2A.X, and Draq5 in sections of human young and old tumor nephrectomy kidneys with quantification of gH2A.X foci per podocyte nuclear area and per podocyte nucleus, scalebar indicating 5 μm, (unpaired t-test, n≥4, 5 glomeruli per sample, 5 podocytes per glomerulus).

E: Representative immunofluorescence staining of synaptopodin (SNP, gray), DNA damage signaling kinase phospho-DNA PKc (green) and nuclear marker DAPI (red) in sections of 14-week-old versus 96-week-old wildtype (WT) mice kidneys, with quantification of SNP cells with pDNA PKc positive nuclei per total SNP positive cells. Yellow circles indicating positive nuclei, red circles indicating negative nuclei, scalebar indicating 50 μm (unpaired t-test, n=4, 10 glomeruli per sample).

F: Representative immunofluorescence staining of synaptopodin (SNP, gray), DNA damage signaling kinase phospho-ATM (green) and nuclear marker DAPI (red) in sections of 14-week-old versus 96-week-old wildtype (WT) mice kidneys, with quantification of SNP cells with pATM positive nuclei per total SNP positive cells. Yellow circles indicating positive nuclei, red circles indicating negative nuclei, scalebar indicating 10 μm (unpaired t-test, n=4, 10 glomeruli per sample).

Violin plots indicating median (black) and upper and lower quartile (gray), *p ≤ 0,05, **p ≤ 0,01, ***p ≤ 0,001, ****p ≤ 0,0001.

Figure S6: The glomerular expression of DNA repair genes is altered in different kidney diseases

Expression profile of 118 hallmark DNA repair genes in MCD, FSGS, IgA nephropathy (IgA), membranous nephropathy (MGN), diabetic nephropathy (DN), and hypertension (HT) glomeruli compared to controls depicted as parts of whole and single genes in heatmaps. Genes ranked by their protein abundance (Intensity-based absolute quantification - IBAQ) in murine podocyte proteome analysis (1).

Figure S7: Podocyte specific expression of ERCC family and RNA polymerase subunits obtained through single nucleus sequencing of patient biopsies.
Scatterplots of ERCC family and RNA polymerase subunit expression data of single podocytes obtained from living donor (LD), Minimal change disease (MCD), and FSGS biopsies (2).

Table S1: Gene expression analysis of ERCB for Hallmark DNA Repair and Nucleotide Excision Repair Genes

Table S2: Clinical characteristics of FSGS patients

Table S3: eQTL analysis of DNA repair genes in FSGS patients
Ensg: ensemble gene ID; FDR: false discovery rate, PIP: posterior inclusion probability, AF: allele frequency, beta: expression difference to reference allele

Table S4: Full eQTL analysis of DNA repair genes in FSGS patients

Supplementary Methods:
Loss of genome maintenance is linked to mTORC1 signaling and accelerates podocyte damage

GSEA Analysis

Raw data were downloaded from GSE43061, normalized with RMA (3), and further processed with limma (4). The log fold-changes of the comparison *Ercc1* 14 weeks vs. WT 14 weeks were used as input for the gene length GSEA analysis (5). The p-values and adjusted p-values are depicted in the plots. The enrichment analysis for chromosomal gene distributions was done in R v3.6.3 with the GSEA function of clusterProfiler (6), v3.14.3 was used with maxGSSize=6000 and nPerm=20000.

Mice

Mice were bred in a mixed FVB/CD1 (*Ercc1* pko) or FVB/CD1/C57BL/6 (*Ercc1* ipko) background. All offspring was born in normal mendelian ratios. Mice were housed in the animal facility of the Center for Molecular Medicine Cologne or the Cluster of Excellence – Cellular Stress Responses in Aging-Associated Diseases. Following federal regulations, the Animal Care Committee of the University of Cologne reviewed and approved the experimental protocols. Animals were housed at specific pathogen-free (SPF) conditions with three-monthly monitoring according to FELASA suggestions. Housing was done in groups of less than six

adult animals receiving CRM pelleted breeder and maintenance diet irradiated with 25 kGy (Special Diet Services, Witham, UK) and water *ad libitum*. Spot urine was collected once a week during cage changes or during sacrifice. Tamoxifen was administered at 400 mg/kg Tamoxifen in dry chow starting when mice were 8 weeks of age for a total of 4 weeks.

For rapamycin injection studies male and female *Ercc1*$^{fl/fl}$ (ctrl) and *Ercc1*; *Nphs2.Cre* (pko) mice at week 6 of age were injected intraperitoneally 3 times/week with 2 mg/kg bodyweight of rapamycin diluted in 5% ethanol, 5% tween 80, and 5% PEG 400 or with 5% ethanol, 5% tween 80, and 5% PEG 400 as vehicle. Urine collection was performed 2 times/week and mice were sacrificed at week 13 of age for serum and kidney tissue isolation. All animals were maintained in grouped cages on a 12h light/dark cycle. Mice were kept on a regular diet and had access to water *ad libitum*. Body weight was measured weekly. Animals were housed in a temperature-controlled, pathogen-free animal facility at the Institute of Molecular Biology and Biotechnology (IMBB), which operates in compliance with the “Animal Welfare Act” of the Greek government, using the “Guide for the Care and Use of Laboratory Animals” as its standard.

Mice were anaesthetized by intraperitoneal injection of 10 μl per g bodyweight of 0.01% xylocaine and 12.5 mg/ml ketamine – blood was drawn from the left ventricle into a syringe rinsed with Heparin sulfate and animals were perfused with cold phosphate buffered saline (PBS). Kidneys were excised and embedded in OCT (Sakura, Torrance, CA) and frozen at -80°C or fixed in 4% neutral buffered formalin and subsequently embedded in paraffin.

Archival tissue of podocyte specific *Tsc*1 knockout mice was provided by Tillman Bork (University of Freiburg, Germany). *Tsc1*$^{fl/fl}$ mice were crossbred with Nphs2-Cre^{+}. For further details see ref (7).

Tissue of *Ercc1* -/delta mice was provided by Martijn Dollé (National Institute of Public Health and the Environment, Bilthoven, Netherlands). Further details provided in ref (8).

Electron Microscopy

Mice were perfused with 4% paraformaldehyde and 2% glutaraldehyde in 0.1 M sodium cacodylate, pH 7.4. Postfixation was performed in the same buffer for two additional weeks at 4°C. Tissue was osmicated with 1% OsO4 in 0.1 M cacodylate and dehydrated in increasing ethanol concentrations. Upon infiltration and flat embedding were performed following standard procedures. Toluidine blue was used to stain semithin sections of 0.5 μm. 30 nm-thick sections were cut with an Ultracut UCT ultramicrotome (Reichert) and stained with 1% aqueous uranylic acetate and lead citrate. Samples were studied with Zeiss EM 902 and Zeiss EM 109 electron microscopes (Zeiss, Oberkochen, Germany).

Podocyte isolation

To isolate primary podocytes, *Ercc1*$^{fl/fl}$ mice heterozygous for the *R26mTmG* and *Nphs2.Cre* transgene were sacrificed and kidneys were used for glomerular preparation, as previously described (1). The glomeruli were digested and the single-cell suspension was used for fluorescence-activated cell sorting.

qPCR Analysis

Total ribonucleic acid (RNA) was extracted from podocytes of *Ercc1/Nphs2.Cre/mTmG* mice using Direct-zol™ RNA MiniPrep Kit (cat. no. R2052, Zymo Research). Isolation of glomeruli, preparation of a glomerular single-cell suspension, and fluorescence-activated cell sorting was done as previously described (9). Podocytes were sorted into TriReagent (cat. no. 93289, Sigma-Aldrich). The complementary deoxyribonucleic acid (cDNA) was synthesized with High Capacity cDNA Reverse Transcription Kit (cat. no. 4368814, Applied Biosystems). PCR was performed using TaqMan™ Gene Expression Master Mix (cat. no. 4369016, Applied Biosystems) and the Applied Biosystems Real-time PCR system. Real-time PCR was measured with triplicates in each gene target. The sequence of the PCR primer used for *Ercc1* was: 5′- AGCCAGACCCTGAAAACAG-3′ and 5′- CACCTCACCGAATTCCCA-3′ in PrimeTime Mini qPCR Assay for *Ercc1* (Assay-ID: Mm.PT.58.42152282, IDT). The gene expression was calculated using comparative cycle threshold method and normalized to RNA polymerase II subunit A (*Polr2a*). The relative fold change of *Ercc1* expression in knockout mice was compared with WT and heterozygous mice.

Urinary Albumin ELISA & Creatinine measurement

Urinary albumin levels were measured with a mouse albumin ELISA kit (mouse albumin ELISA kit; Bethyl Labs, Montgomery, TX, USA). Urinary creatinine kit (Cayman Chemical, Ann Arbor, MI, USA) was used to determine corresponding urinary creatinine values. For Coomassie Blue detection of albuminuria, spot urine of mice was diluted 1:20 in 1x Laemmli buffer and urinary proteins separated using poly-acrylamide gel electrophoresis with subsequent Coomassie gel stain.

Plasma Creatinine and Urea measurement

Blood samples were centrifuged at 400 g 4°C for 20 minutes and plasma samples subsequently stored at -20°C until further analysis. Creatinine and Urea were measured using standard clinical protocols by the Department of Clinical Chemistry of the University of Cologne.

Histologic analysis

To assess morphological changes in light microscopy we performed Periodic Acid Schiff staining.

Immunofluorescence Staining

Paraffin embedded tissue was cut into 3 μm thick sections and processed according to published protocols (10). Primary antibodies (anti-γH2A.X #2577s – Cell Signaling Technology, anti-nephrin #GP-N2 – Progen, anti-synaptopodin #65294 – Progen, anti-Dach1 #HPA012672 – Sigma Aldrich (11), anti-phospho-S6 Ribosomal Protein (Ser235/236) # 4858 – Cell Signaling Technology, anti-phospho-STING (Ser336) # 19781 – Cell Signaling Technology, anti-phospho-PKC alpha (Ser657) # sc-12356 – Santa Cruz Biotechnology and anti-p53 #p53-protein-cm5 Leica Biosystems, anti-ATM (phospho S1987) antibody [EPR28058-71] # ab315019 – abcam, anti-DNA PKcs (phospho S2056) antibody - ChIP Grade # ab18192 – abcam) were used at 1:200 dilution. DAPI or Far-red fluorescent DNA dye Draq 5 was used as a nuclear marker.

Cells were processed according to published protocols (12).

For γH2A.X foci quantification, a custom-built FIJI macro was used. In brief, podocyte nuclei were identified through surrounding synaptopodin staining, segmented using the freehand tool and split into single channels. Draq 5 channel was converted into binary image using auto threshold "otsu dark" with subsequent particle measurement (range 5-infinite) to determine nuclear area. γH2A.X channel was converted into binary image using auto threshold "MaxEntropy dark" with subsequent particle measurement (range 0.02-infinite) to determine foci number and area.

Glomerular isolation and RNA sequencing analysis

Glomerular isolation was performed according to the previously described protocol (1) and flash frozen in liquid nitrogen for storage. Glomeruli were lysed in Trizol and total RNA was extracted using the Direct-zol RNA Miniprep kit (Zymo) according to manufacturer's instructions. Libraries were prepared from 500ng total RNA. Enzymatic depletion of ribosomal RNA with the Illumina Ribo-Zero Plus rRNA Depletion Kit was followed by library preparation with the Illumina® Stranded Total RNA sample preparation kit. The depleted RNA was fragmented and reverse-transcribed with random hexamer primers, and second-strand synthesis with dUTPs was followed by A-tailing, adapter ligation, and library amplification (12 cycles). Next library validation and quantification (Agilent Tape Station) was performed, followed by pooling of equimolar amounts of library. The pool itself was then quantified using the Peqlab KAPA Library Quantification Kit and the Applied Biosystems 7900HT Sequence Detection System and sequenced on an Illumina NovaSeq6000 sequencing instrument with a PE100 protocol aiming for 30 million clusters per sample.

Transcript quantification was performed using Salmon (v1.9)(13) with the parameters --validateMappings, --seqBias, and --gcBias, employing a decoy-aware reference transcriptome based on GRCm39 and a k-mer size of 31 (-k 31). Transcript-level quantifications were summarized to the gene level using tximport (v1.14.2)(14) . Lowly expressed genes were

filtered using the filterByExpr function from the edgeR package (v3.40.2)(15) . Differential expression analysis was subsequently performed using edgeR.

To assess gene length effects, log_{10}-transformed gene lengths were calculated and compared between significantly upregulated and downregulated genes (FDR < 0.05) using Seaborn-0.11.0's kdeplot function(16) in Python for density visualization.

For comparison with published datasets, raw gene count files (GSE244072_KSP_gene_count.txt.gz and GSE244072_KSP_DMSO_RAPA_gene_count.txt.gz) were downloaded from the GEO database (accession: GSE244072)(17) . Differential gene expression analysis of these datasets was carried out using edgeR as described above.

KEGG pathway enrichment analysis was performed using the fgseaMultilevel function(18) from the clusterProfiler package (v4.9.2.002)(19) with nPerm = 20,000.

The RNA-seq data are available from the Gene Expression Omnibus (GEO) under the accession number GSE292420.

In vitro Experiments

Conditional immortalized murine podocytes were a gift by Stuart Shankland. Cells were cultured as previously described (20). Briefly, immortalized podocytes were cultured in RPMI media supplemented with 10% FBS and IFNγ (Sigma-Aldrich, Taufkirchen, Germany). Cells proliferated at 33°C on Primaria plastic plates (BD Biosciences, San Jose, CA, USA) until they reached a confluence of 60-70%. Differentiation of podocytes was induced by seeding the cells at 37°C in the absence of IFNy. After 10 days of differentiation, cells were treated with 5 or 10 μg/ml Mitomycin C (#M0503 - Sigma-Aldrich, Taufkirchen, Germany) for 2h in serum-free medium, followed by one washing step with PBS and another 6h incubation in serum-free medium without Mitomycin C before further processing. The absence of mycoplasm infection was tested regularly using the mycoplasm detection kit from Minerva biolabs (Minerva Biolabs, Berlin, Germany). For experiments with DNA damage response inhibitors, differentiated cells were pre-treated with 3 μM KU60019 (Selleckchem, Houston, TX, USA) or 1 μM Nedisertib (Selleckchem, Houston, TX, USA) for 1 h before inducing DNA damage by UV-C or Mitomycin C treatment. Inhibitors were added again after medium change following DNA damage induction to further incubate cells for 6 h before cell lysis.

Western Blot analysis

SDS-PAGE was used for protein size separation with subsequent blotting onto polyvinylidene difluoride membranes and visualized with enhanced chemiluminescence after incubation of the blots with corresponding antibodies (Phospho-Histone H2A.X (Ser139) #2577s; Phospho-S6 Ribosomal Protein (Ser235/236) #4858; S6 Ribosomal Protein (5G10) #2217 – Cell

Signaling Technology; alpha Actin (JLA20) – Developmental Studies Hybridoma Bank; beta-Tubulin (E7) - Developmental Studies Hybridoma Bank).

Preparation of formalin-fixed paraffin embedded tissue for STED microscopy

Kidney tissue samples were prepared according to a previously published protocol, with slight modifications(21). 60 μm sections were cut from paraffin blocks using a microtome. Sections were then transferred to 1.5 mL Eppendorf tubes and de-paraffinized according to standard procedures using xylene with re-hydration in decreasing concentrations of ethanol in DI water. After this, sections were cleared in clearing solution (CS) (200 mM boric acid, 4% SDS, pH 8.5) for 6 hours at 70°C, before proceeding with immunolabelling after washing in PBST for 3*5 minutes.

Immunolabelling

Samples were incubated in a sheep polyclonal antibody to nephrin (R&D systems, AF4269) diluted at 1:50 in 10 mM HEPES pH 7.5 with 200 mM NaCL and 10% TritonX-100 (HEPES-TCS buffer) at 37°C for 24 hours with shaking at 500 rpm. Samples were washed in PBST for 3*5 min and were then incubated in a donkey anti-sheep secondary antibody conjugated to Abberior STAR635P (Abberior, 2-0142-007-2, dilution 1:50) at 37°C for 24 hours.

Mounting and Imaging

Samples were incubated in 80% wt/wt fructose (1 mL of dH_20 added to 4 g of fructose) at 37°C with shaking at 500 rpm for 15 minutes and then placed in a MatTek dish with a cover slip on top (to prevent evaporation) prior to imaging. Images were acquired using a Leica SP8 3X gSTED system using a 100X 1.4 NA objective.

Post-processing and Quantitative Analysis

Prior to quantitative analysis, all images were deconvolved using SVI Huygens software. The slit diaphragm (SD) length was measured using an ImageJ macro as previously(22).

ERCB Human microarray analysis

167 genes involved in DNA repair and nucleotide excision repair were compiled from the hallmark gene set “DNA-Repair” from the Molecular Signatures Database (MSigDB) Collection (23) and upon literature research. Human kidney biopsies and Affymetrix microarray expression data were obtained within the framework of the European Renal cDNA Bank - Kröner-Fresenius Biopsy Bank (24). Diagnostic biopsies were obtained from patients after informed consent and with approval of the local ethics committees. Following renal biopsy, the tissue was transferred to RNase inhibitor and micro-dissected into glomeruli and tubulo-interstitium. Total RNA was isolated, reverse transcribed, and amplified as previously reported (25). Fragmentation, hybridization, staining, and imaging were performed according to the Affymetrix Expression Analysis Technical Manual (Affymetrix, Santa Clara, CA, USA). Published datasets of glomerular samples were analysed for mRNA expression levels.

Analysis included datasets from patients with minimal change disease (MCD; n=14), focal segmental glomerulosclerosis (FSGS; n=23), membranous nephropathy (MGN; n=21), IgA nephropathy (Glom; n=27), and hypertensive nephropathy (HTN; n=15) as well as controls (living donors (LD); n=42) (GSE99340, LD data from: GSE32591, GSE37463). CEL file normalization was performed with the Robust Multichip Average method using RMAExpress (Version 1.0.5) and the human Entrez-Gene custom CDF annotation from Brain Array version 18 (http://brainarray.mbni.med.umich.edu/Brainarray/default.asp). To identify differentially expressed genes, the SAM (Significance Analysis of Microarrays) method (26) was applied using SAM function in Multiple Experiment Viewer (TiGR MeV, Version 4.9). A q-value below 5% was considered to be statistically significant. The resulting gene expression list was censored for genes, whose products were detected in a transcriptomic and proteomic analysis of wild-type murine podocytes (1).

Single nucleus sequencing

Nuclei were prepared from kidney biopsy cores stored in RNAlater from FSGS patients enrolled in the NEPTUNE study (2). The processing followed the protocol developed from the Kidney Precision Medicine Project. Nuclei preparations were processed and sequenced using 10x Genomics single cell sequencer. Analyses were performed on the output data files from CellRanger v6.0.0 using the Seurat R package (version 3.2 and 4.0; https://cran.r-project.org/web/packages/Seurat/index.html). To limit low quality nuclei and/or multiplets, we set gene counts and cutoffs to between 500 and 5000 genes and examined nuclei with a mitochondrial gene content of less than 10%. Nuclei were merged into a Seurat object using the CCA integrate function and nuclear cluster annotation was determined by finding enriched genes in each cell cluster. A comparison of these cluster selective gene profiles was compared against previously identified cell marker gene sets from human kidney samples from KPMP and other sources (2).

Expression quantitative trait locus (eQTL) analysis

For the subgroup analysis of FSGS cohort, the procedure described in Gillies et al., 2018 was used with the following exceptions: only FSGS patients were analysed (N=87) and only RNAseq expression data for glomerular samples were utilized. Briefly, cis-eQTLs were identified using MatrixEQTL from among variants that were located either within the annotated boundaries of a gene or its surrounding region (+/- 500 kb) (27). We then adjusted for age, sex, principal components of genetic ancestry, and the first 5 PEER factors (28). The genetic ancestry was calculated using LD-pruned WGS data from across all 87 patients using the EPACTS tool (https://genome.sph.umich.edu/wiki/EPACTS). The gene-level FDR for the MatrixEQTL was controlled using TORUS. Fine mapping of the eQTLs was performed using the DAP algorithm (29).

Study approval

All investigations involving human specimen have been conducted according to the Declaration of Helsinki following approval of the local ethics committees. Written informed consent was received from participants prior to inclusion in the study. All mouse experiments were conducted according to institutional and federal guidelines and approved by the LANUV NRW VSG 84-02.04.2013.A336.

Statistics

If not stated otherwise, unpaired two tailed Student's t-test was used to compare two groups and p-values ≤ 0.05 were considered significant. For multiple group comparisons, we applied 1-way ANOVA or 2-way ANOVA where applicable followed by Tukey's post hoc correction. Statistics were performed using GraphPad Prism 8-10.

Sex as a biological variable

Our study examined male and female animals, and similar findings are reported for both sexes.

Data availability

All expression data is available through Gene Expression Omnibus under the Accession numbers:

GSE43061

GSE244072

GSE292420

GSE99340

GSE32591

GSE37463

GSE213030

All additional data displayed in the figures is provided in the file: Single Values 172370-INS-RG-RV-3 provided to the editorial office.

References:

1. Rinschen MM, et al. A Multi-layered Quantitative In Vivo Expression Atlas of the Podocyte Unravels Kidney Disease Candidate Genes. *Cell reports*. 2018;23(8):2495–2508.

2. Mariani LH, et al. Precision nephrology identified tumor necrosis factor activation variability in minimal change disease and focal segmental glomerulosclerosis. *Kidney Int*. [published online ahead of print: 2022]. https://doi.org/10.1016/j.kint.2022.10.023.

3. Irizarry RA, et al. Exploration, normalization, and summaries of high density oligonucleotide array probe level data. *Biostatistics (Oxford, England)*. 2003;4(2):249–264.

4. Ritchie ME, et al. limma powers differential expression analyses for RNA-sequencing and microarray studies. *Nucleic Acids Res*. 2015;43(7):e47–e47.

5. Schermer B, et al. Transcriptional profiling reveals progeroid Ercc1-/Δ mice as a model system for glomerular aging. *Bmc Genomics*. 2013;14(1):559.

6. Yu G, et al. clusterProfiler: an R Package for Comparing Biological Themes Among Gene Clusters. *Omics J Integr Biology*. 2012;16(5):284–287.

7. Bork T, et al. Podocytes maintain high basal levels of autophagy independent of mtor signaling. *Autophagy*. 2020;16(11):1932–1948.

8. Birkisdóttir MB, et al. Unlike dietary restriction, rapamycin fails to extend lifespan and reduce transcription stress in progeroid DNA repair-deficient mice. *Aging Cell*. 2021;20(2):e13302.

9. Wanner N, et al. Unraveling the role of podocyte turnover in glomerular aging and injury. *Journal of the American Society of Nephrology : JASN*. 2014;25(4):707–716.

10. Puelles VG, et al. Podocyte Number in Children and Adults: Associations with Glomerular Size and Numbers of Other Glomerular Resident Cells. *J Am Soc Nephrol*. 2015;26(9):2277–2288.

11. Endlich N, et al. The transcription factor Dach1 is essential for podocyte function. *J Cell Mol Med*. 2018;22(5):2656–2669.

12. Liebau MC, et al. Dysregulated Autophagy Contributes to Podocyte Damage in Fabry's Disease. *Plos One*. 2013;8(5):e63506.

13. Patro R, et al. Salmon provides fast and bias-aware quantification of transcript expression. *Nat Methods*. 2017;14(4):417–419.

14. Soneson C, Love MI, Robinson MD. Differential analyses for RNA-seq: transcript-level estimates improve gene-level inferences. *F1000Research*. 2016;4:1521.

15. Robinson MD, McCarthy DJ, Smyth GK. edgeR: a Bioconductor package for differential expression analysis of digital gene expression data. *Bioinformatics*. 2009;26(1):139–140.

16. Waskom M. seaborn: statistical data visualization. *J Open Source Softw*. 2021;6(60):3021.

17. Alesi N, et al. TFEB drives mTORC1 hyperactivation and kidney disease in Tuberous Sclerosis Complex. *Nat Commun*. 2024;15(1):406.

18. Korotkevich G, et al. Fast gene set enrichment analysis. *bioRxiv*. 2021;060012.

19. Wu T, et al. clusterProfiler 4.0: A universal enrichment tool for interpreting omics data. *Innov*. 2021;2(3):100141.

20. Shankland SJ, et al. Podocytes in culture: past, present, and future. *Kidney international*. 2007;72(1):26–36.

21. Unnersjö-Jess D, et al. Three-Dimensional Super-Resolved Imaging of Paraffin-Embedded Kidney Samples. *Kidney360*. 2022;3(3):446–454.

22. Butt L, et al. A molecular mechanism explaining albuminuria in kidney disease. *Nat Metabolism*. 2020;2(5):461–474.

23. Liberzon A, et al. The Molecular Signatures Database Hallmark Gene Set Collection. *Cell Syst*. 2015;1(6):417–425.

24. Cohen CD, et al. Quantitative gene expression analysis in renal biopsies: A novel protocol for a high-throughput multicenter application. *Kidney Int*. 2002;61(1):133–140.

25. Cohen CD, et al. Comparative promoter analysis allows de novo identification of specialized cell junction-associated proteins. *Proc National Acad Sci*. 2006;103(15):5682–5687.

26. Tusher VG, Tibshirani R, Chu G. Significance analysis of microarrays applied to the ionizing radiation response. *Proceedings of the National Academy of Sciences of the United States of America*. 2001;98(9):5116–5121.

27. Shabalin AA. Matrix eQTL: ultra fast eQTL analysis via large matrix operations. *Bioinformatics*. 2012;28(10):1353–1358.

28. Stegle O, et al. A Bayesian Framework to Account for Complex Non-Genetic Factors in Gene Expression Levels Greatly Increases Power in eQTL Studies. *Plos Comput Biol*. 2010;6(5):e1000770.

29. Wen X, et al. Efficient Integrative Multi-SNP Association Analysis via Deterministic Approximation of Posteriors. *Am J Hum Genetics*. 2016;98(6):1114–1129.

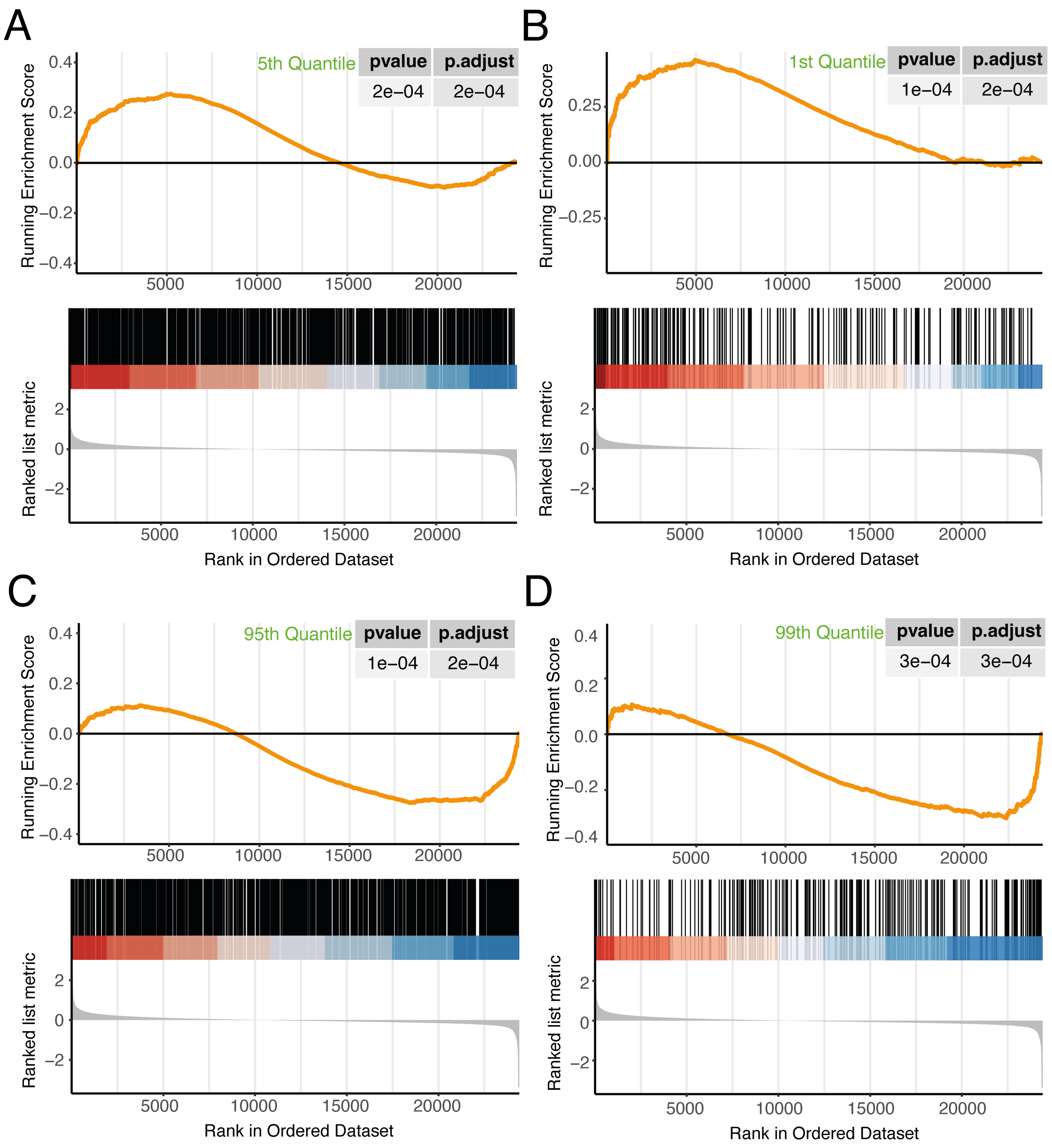
A
5th Quantile
pvalue
p.adjust
2e−04
2e−04
Running Enrichment Score
Ranked list metric
Rank in Ordered Dataset
B
1st Quantile
pvalue
p.adjust
1e−04
2e−04
Running Enrichment Score
Ranked list metric
Rank in Ordered Dataset
C
95th Quantile
pvalue
p.adjust
1e−04
2e−04
Running Enrichment Score
Ranked list metric
Rank in Ordered Dataset
D
99th Quantile
pvalue
p.adjust
3e−04
3e−04
Running Enrichment Score
Ranked list metric
Rank in Ordered Dataset

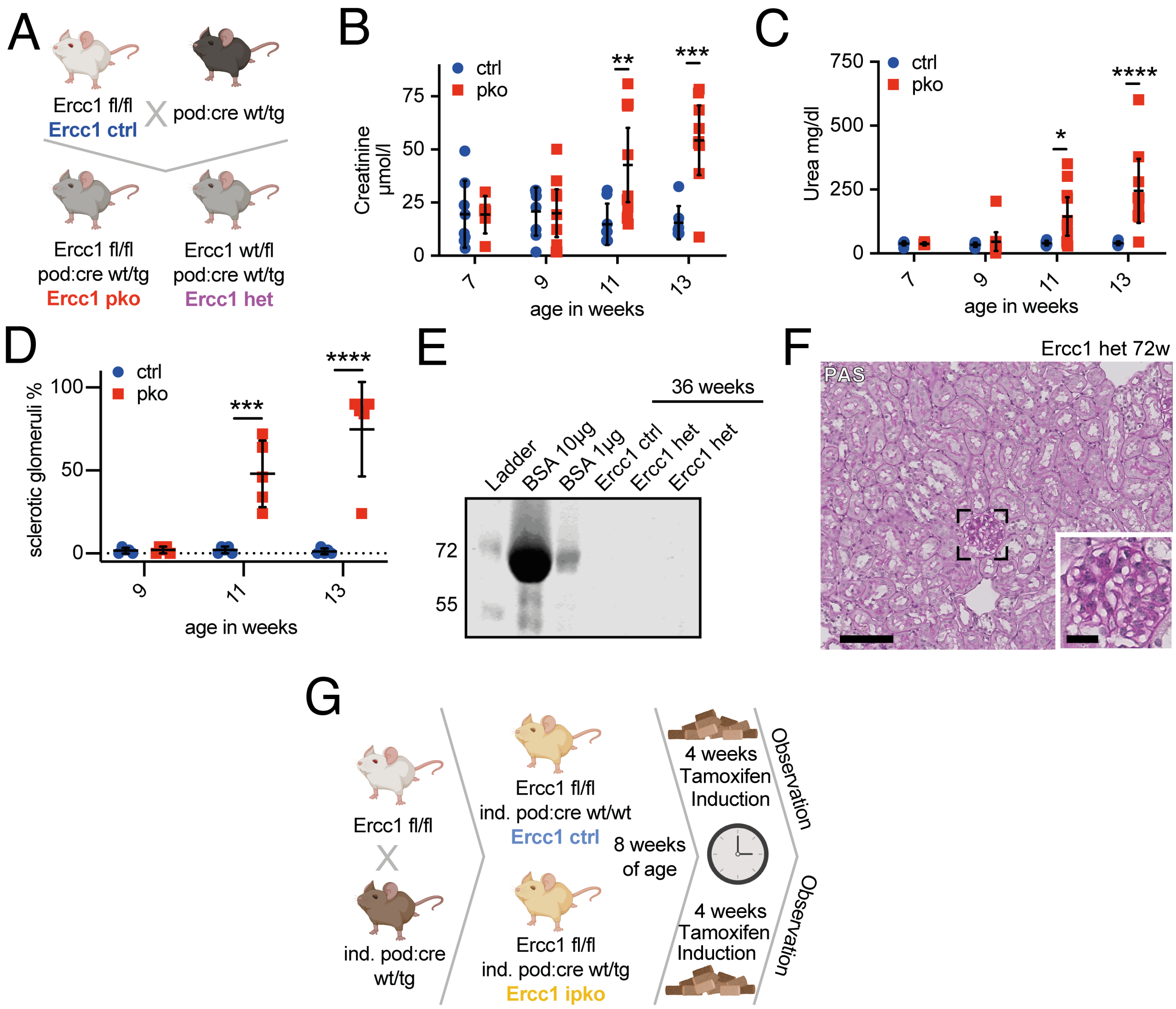
A
Ercc1 fl/fl
Ercc1 ctrl
X
pod:cre wt/tg
Ercc1 fl/fl
pod:cre wt/tg
Ercc1 pko
Ercc1 wt/fl
pod:cre wt/tg
Ercc1 het
B
ctrl
pko
Creatinine µmol/l
**
***
age in weeks
C
ctrl
pko
Urea mg/dl
*
****
age in weeks
D
ctrl
pko
sclerotic glomeruli %
***
****
age in weeks
E
36 weeks
Ladder
BSA 10µg
BSA 1µg
Ercc1 ctrl
Ercc1 het
Ercc1 het
72
55
F
Ercc1 het 72w
PAS
G
Ercc1 fl/fl
X
ind. pod:cre wt/tg
Ercc1 fl/fl
ind. pod:cre wt/wt
Ercc1 ctrl
Ercc1 fl/fl
ind. pod:cre wt/tg
Ercc1 ipko
8 weeks of age
4 weeks Tamoxifen Induction
Observation
4 weeks Tamoxifen Induction
Observation

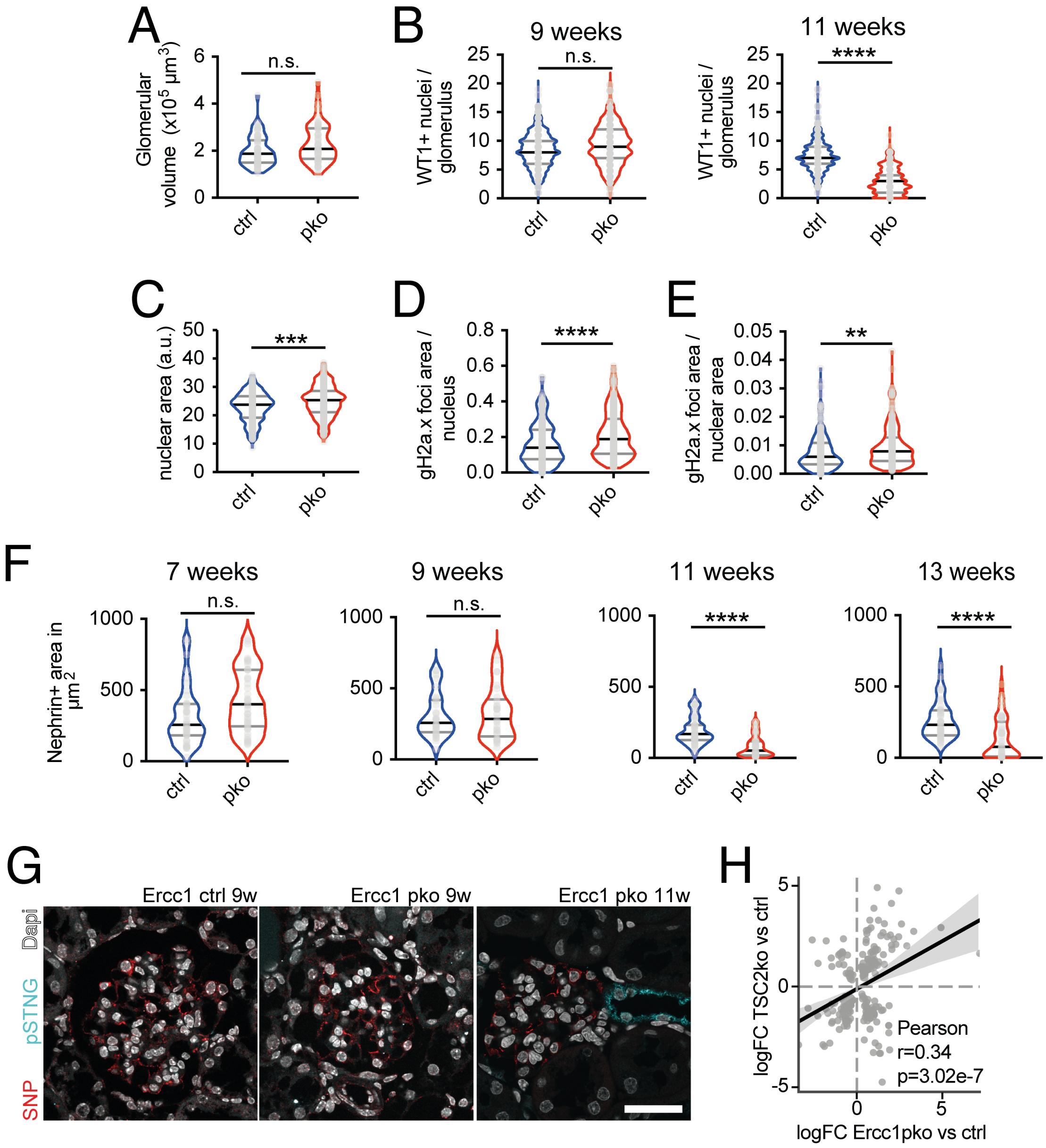

A
Glomerular volume (x10^5 µm^3)
n.s.
ctrl
pko
B
9 weeks
WT1+ nuclei / glomerulus
n.s.
11 weeks
****
C
nuclear area (a.u.)
***
D
gH2a.x foci area / nucleus
****
E
gH2a.x foci area / nuclear area
**
F
7 weeks
9 weeks
11 weeks
13 weeks
Nephrin+ area in µm2
G
Ercc1 ctrl 9w
Ercc1 pko 9w
Ercc1 pko 11w
Dapi
pSTNG
SNP
H
logFC TSC2ko vs ctrl
logFC Ercc1pko vs ctrl
Pearson
r=0.34
p=3.02e-7

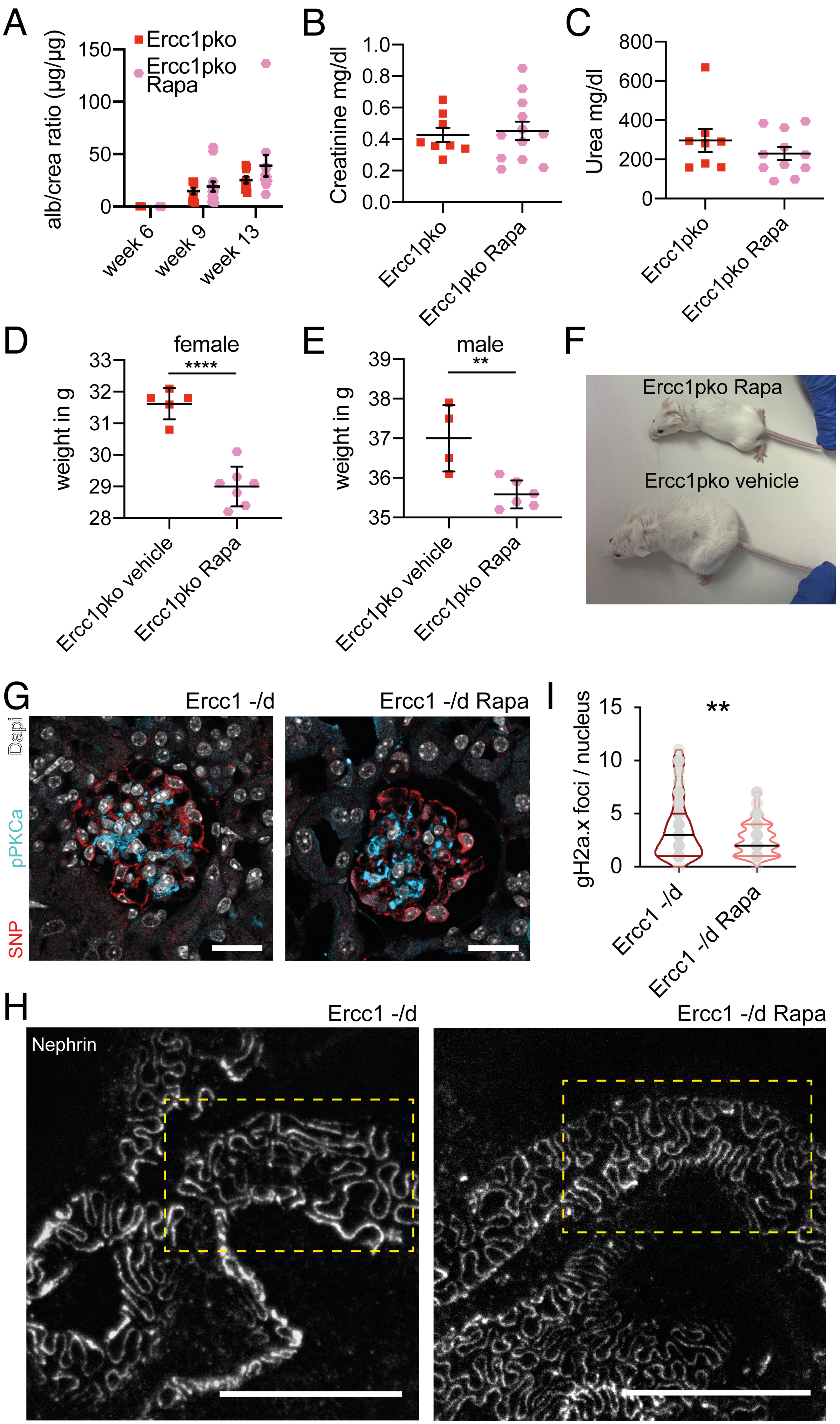

A
Ercc1pko
Ercc1pko Rapa
alb/crea ratio (µg/µg)
150
100
50
0
week 6
week 9
week 13
B
Creatinine mg/dl
1.0
0.8
0.6
0.4
0.2
0.0
Ercc1pko
Ercc1pko Rapa
C
Urea mg/dl
800
600
400
200
0
Ercc1pko
Ercc1pko Rapa
D
female
****
weight in g
33
32
31
30
29
28
Ercc1pko vehicle
Ercc1pko Rapa
E
male
**
weight in g
39
38
37
36
35
Ercc1pko vehicle
Ercc1pko Rapa
F
Ercc1pko Rapa
Ercc1pko vehicle
G
Ercc1 -/d
Ercc1 -/d Rapa
Dapi
pPKCa
SNP
I
**
gH2a.x foci / nucleus
15
10
5
0
Ercc1 -/d
Ercc1 -/d Rapa
H
Ercc1 -/d
Ercc1 -/d Rapa
Nephrin

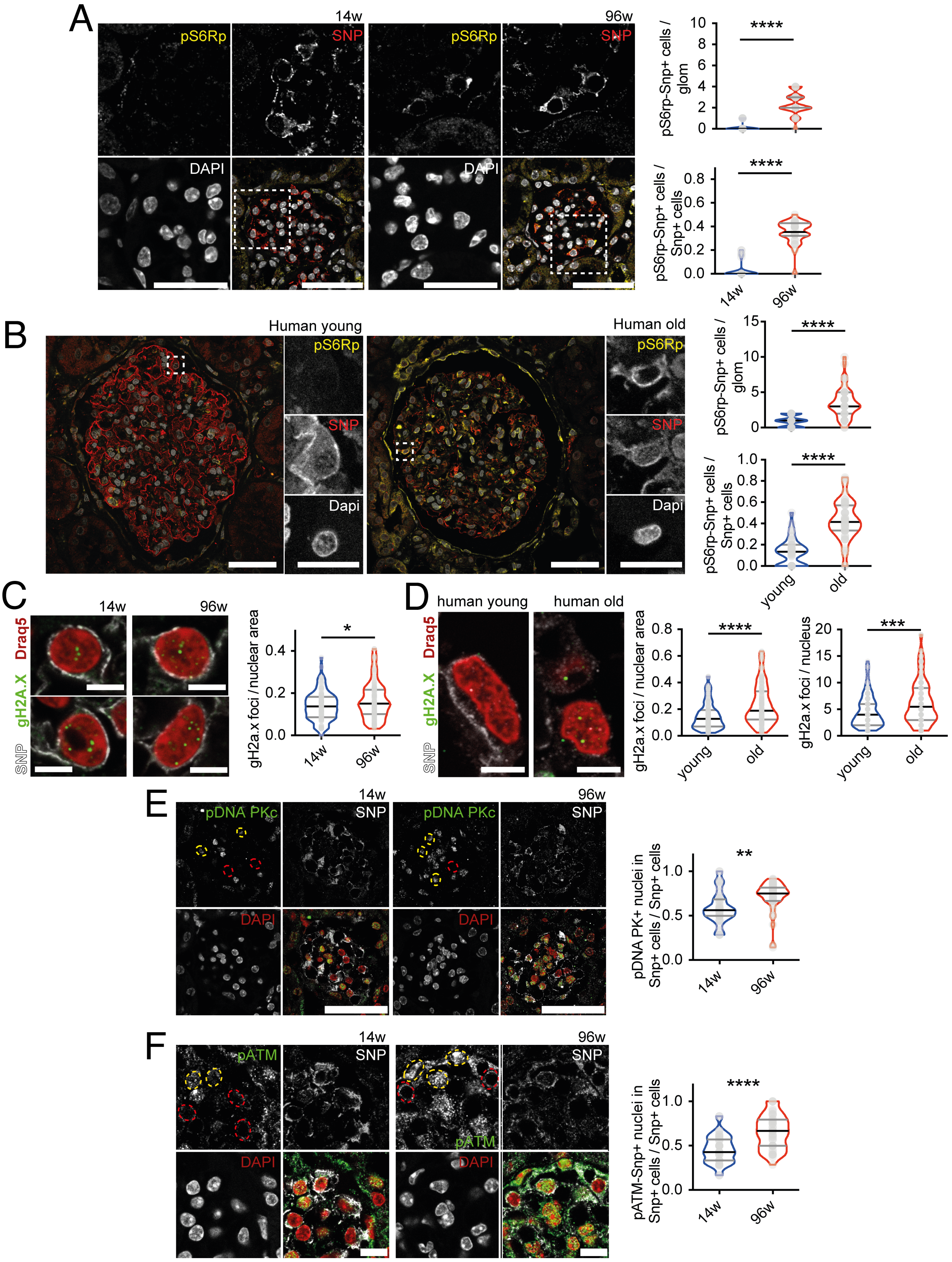
A
14w
96w
pS6Rp
SNP
DAPI
pS6rp-Snp+ cells / glom
****
pS6rp-Snp+ cells / Snp+ cells
14w
96w
B
Human young
Human old
pS6Rp
SNP
Dapi
pS6rp-Snp+ cells / glom
pS6rp-Snp+ cells / Snp+ cells
young
old
C
Draq5
gH2A.X
SNP
gH2a.x foci / nuclear area
*
D
human young
human old
gH2a.x foci / nucleus
***
E
pDNA PKc
pDNA PK+ nuclei in Snp+ cells / Snp+ cells
**
F
pATM
pATM-Snp+ nuclei in Snp+ cells / Snp+ cells

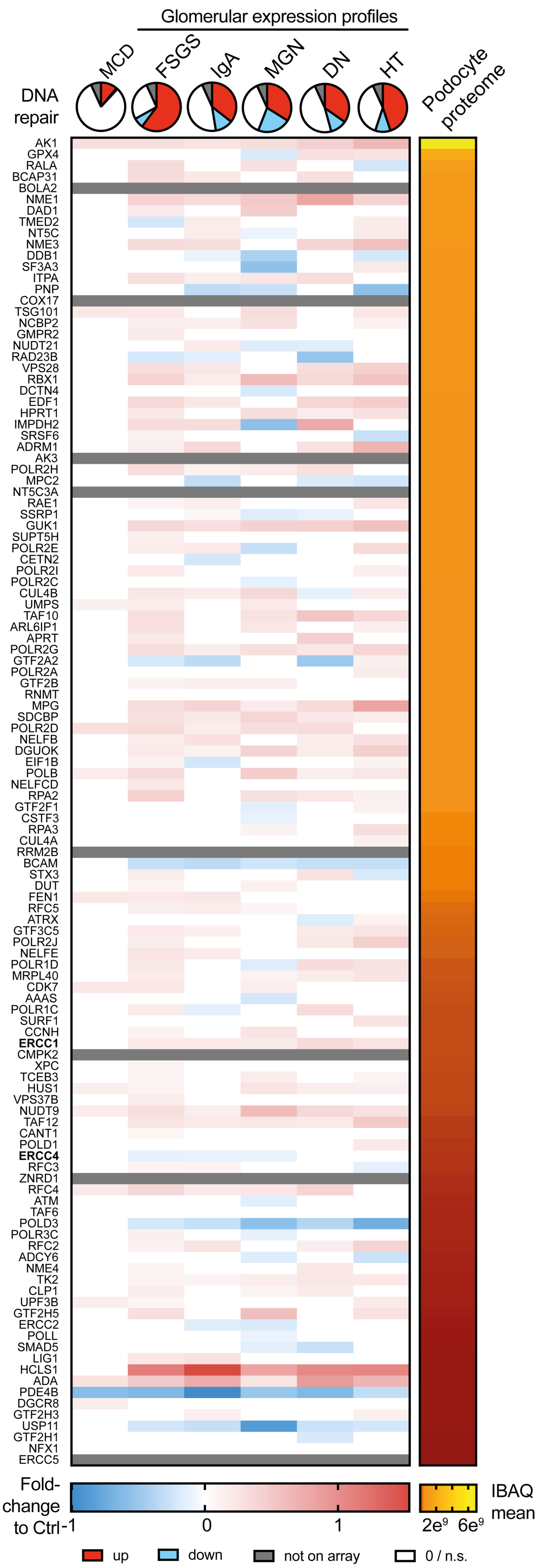

Glomerular expression profiles
MCD
FSGS
IgA
MGN
DN
HT
Podocyte proteome
DNA repair
AK1
GPX4
RALA
BCAP31
BOLA2
NME1
DAD1
TMED2
NT5C
NME3
DDB1
SF3A3
ITPA
PNP
COX17
TSG101
NCBP2
GMPR2
NUDT21
RAD23B
VPS28
RBX1
DCTN4
EDF1
HPRT1
IMPDH2
SRSF6
ADRM1
AK3
POLR2H
MPC2
NT5C3A
RAE1
SSRP1
GUK1
SUPT5H
POLR2E
CETN2
POLR2I
POLR2C
CUL4B
UMPS
TAF10
ARL6IP1
APRT
POLR2G
GTF2A2
POLR2A
GTF2B
RNMT
MPG
SDCBP
POLR2D
NELFB
DGUOK
EIF1B
POLB
NELFCD
RPA2
GTF2F1
CSTF3
RPA3
CUL4A
RRM2B
BCAM
STX3
DUT
FEN1
RFC5
ATRX
GTF3C5
POLR2J
NELFE
POLR1D
MRPL40
CDK7
AAAS
POLR1C
SURF1
CCNH
ERCC1
CMPK2
XPC
TCEB3
HUS1
VPS37B
NUDT9
TAF12
CANT1
POLD1
ERCC4
RFC3
ZNRD1
RFC4
ATM
TAF6
POLD3
POLR3C
RFC2
ADCY6
NME4
TK2
CLP1
UPF3B
GTF2H5
ERCC2
POLL
SMAD5
LIG1
HCLS1
ADA
PDE4B
DGCR8
GTF2H3
USP11
GTF2H1
NFX1
ERCC5
Fold-change to Ctrl
-1
0
1
IBAQ mean
2e9
6e9
up
down
not on array
0 / n.s.

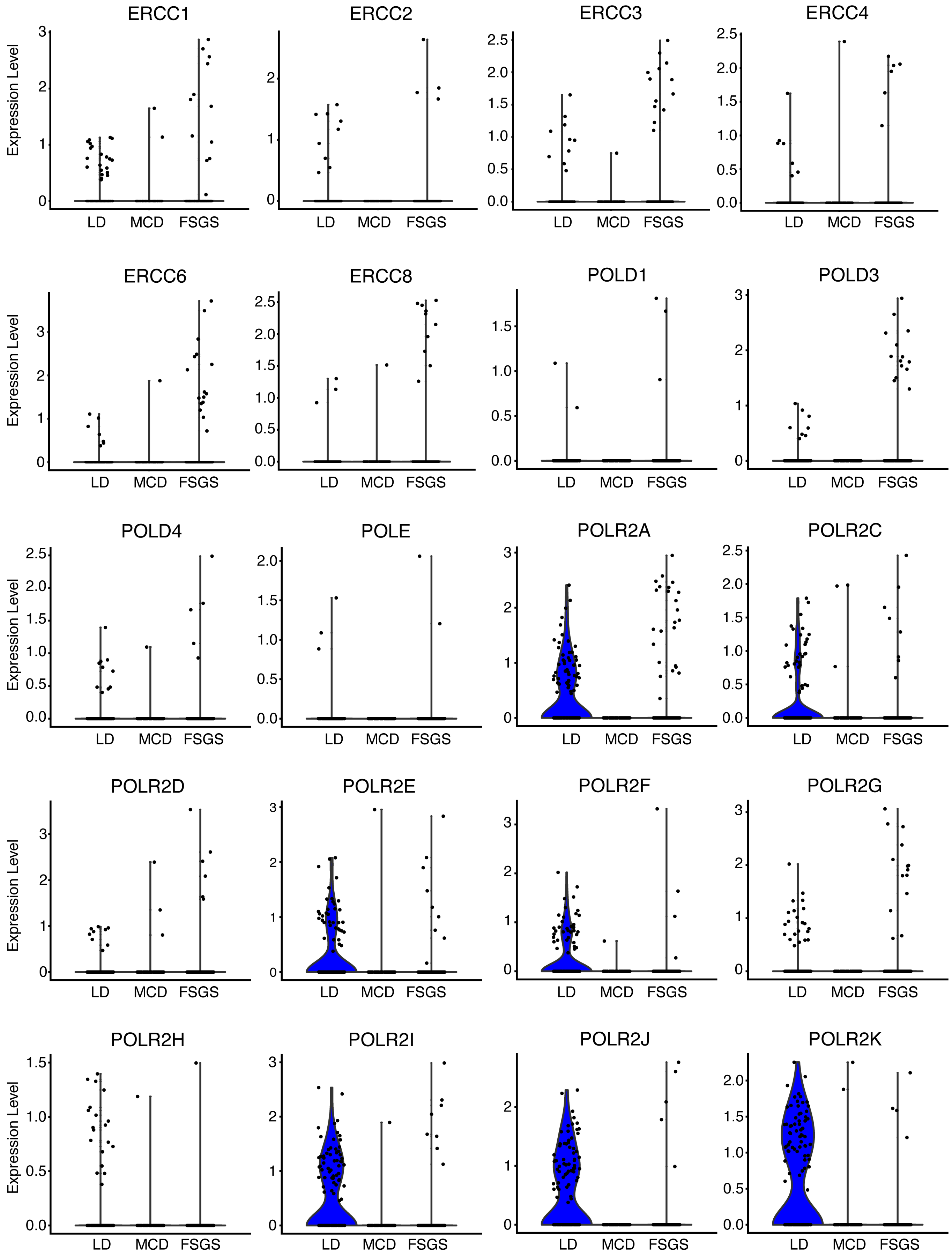

ERCC1
ERCC2
ERCC3
ERCC4
ERCC6
ERCC8
POLD1
POLD3
POLD4
POLE
POLR2A
POLR2C
POLR2D
POLR2E
POLR2F
POLR2G
POLR2H
POLR2I
POLR2J
POLR2K
Expression Level
LD
MCD
FSGS
