# Supplementary material for: Loss of genome maintenance is linked to mTOR complex 1 signaling and accelerates podocyte damage
Source: JCI Insight. 2025 May 20;10(12):e172370. doi: 10.1172/jci.insight.172370 (PMC12220965; doi:10.1172/jci.insight.172370)

Fig. 1H Coomassie

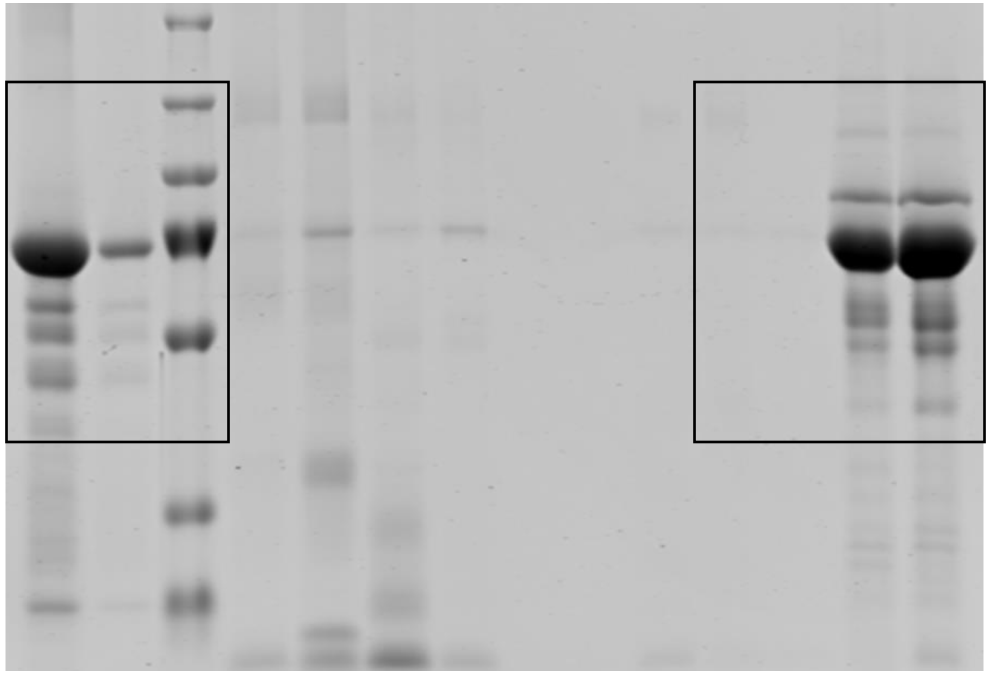

Fig. S2E Coomassie

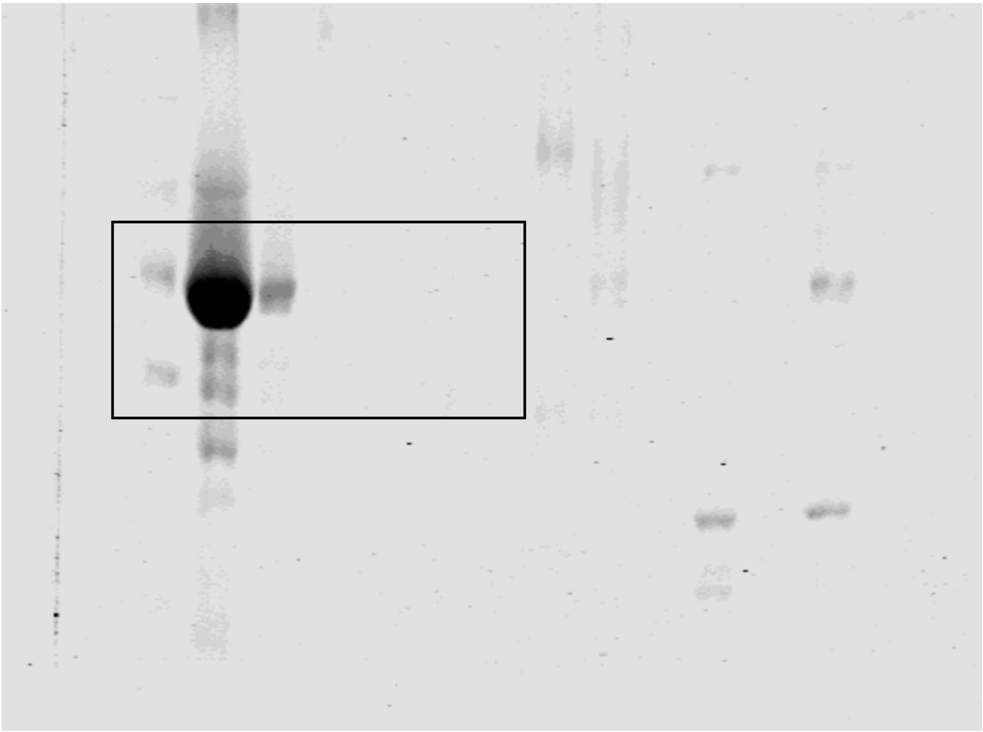

Fig. 4B MMC

pH2A.X

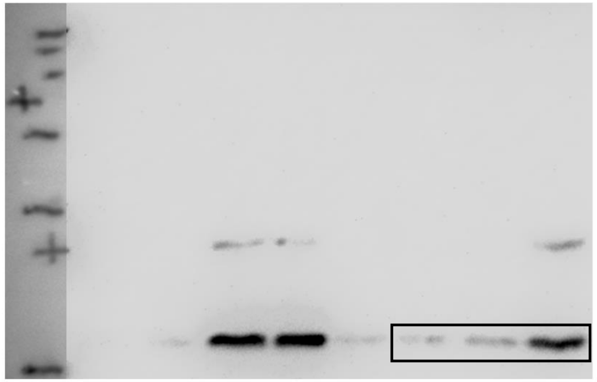

Tubulin

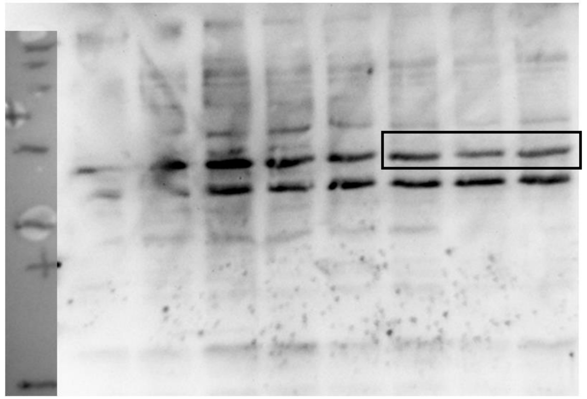

Fig. 4B UV

pH2A.X

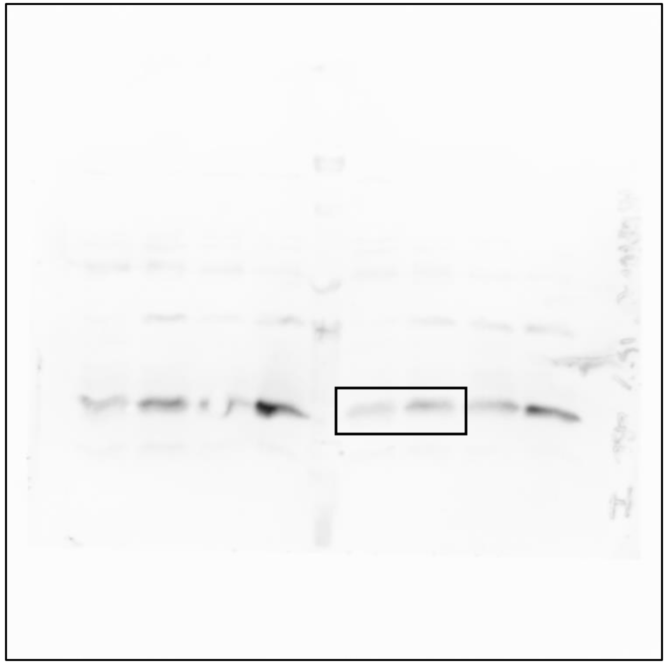

Tubulin

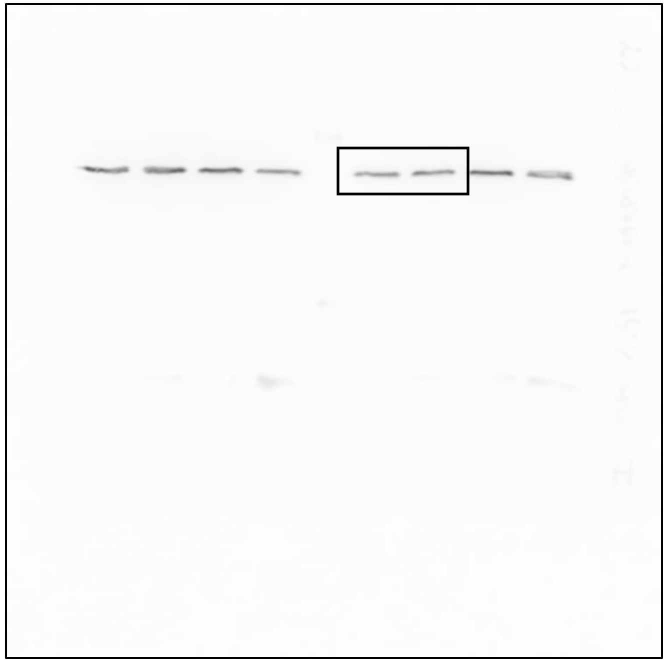

Fig. 4D MMC

pS6RP

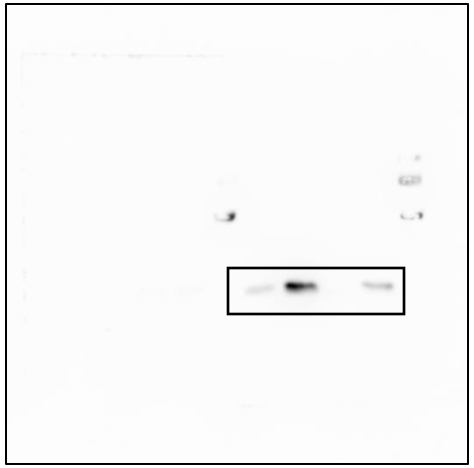

aActin

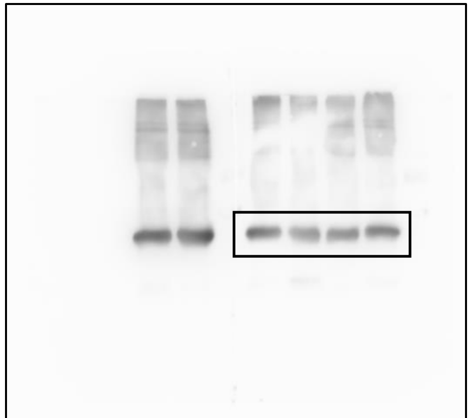

Not depicted in figure

S6RP

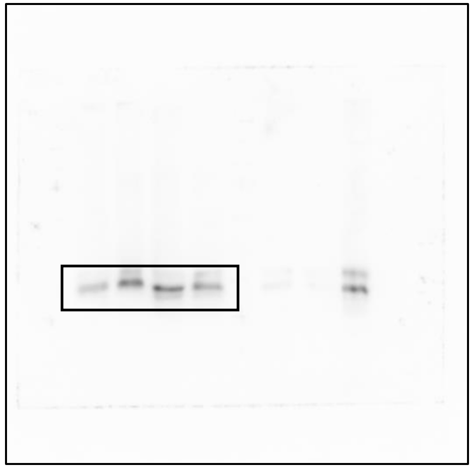

aActin

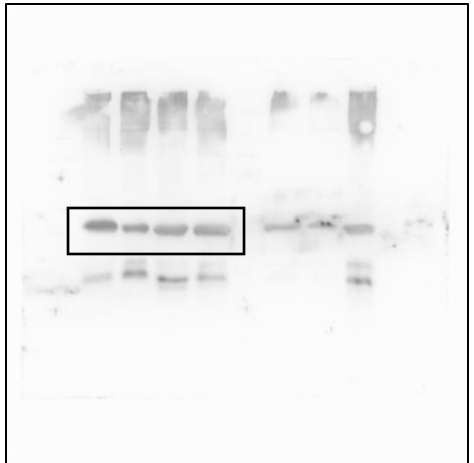

Fig. 4D UV

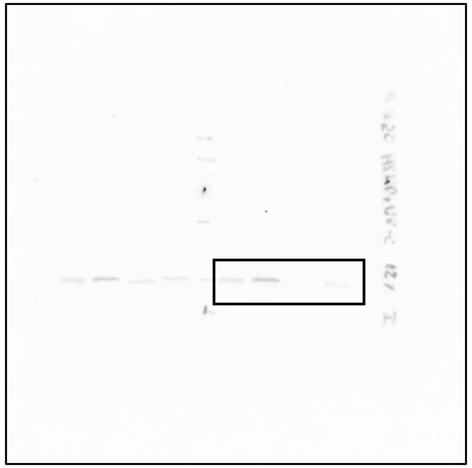

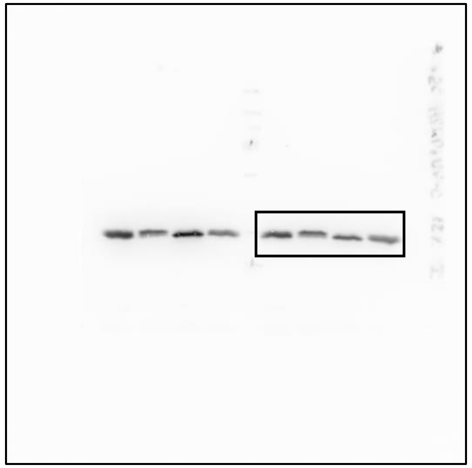

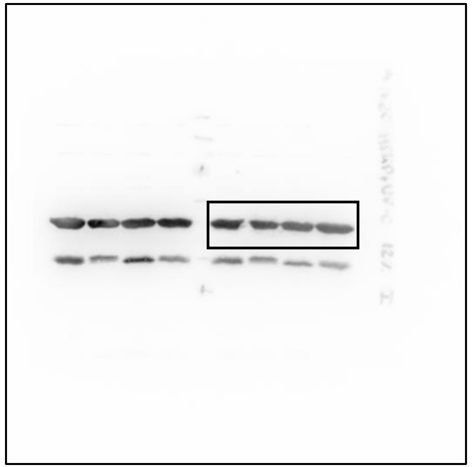

Fig. 4E MMC

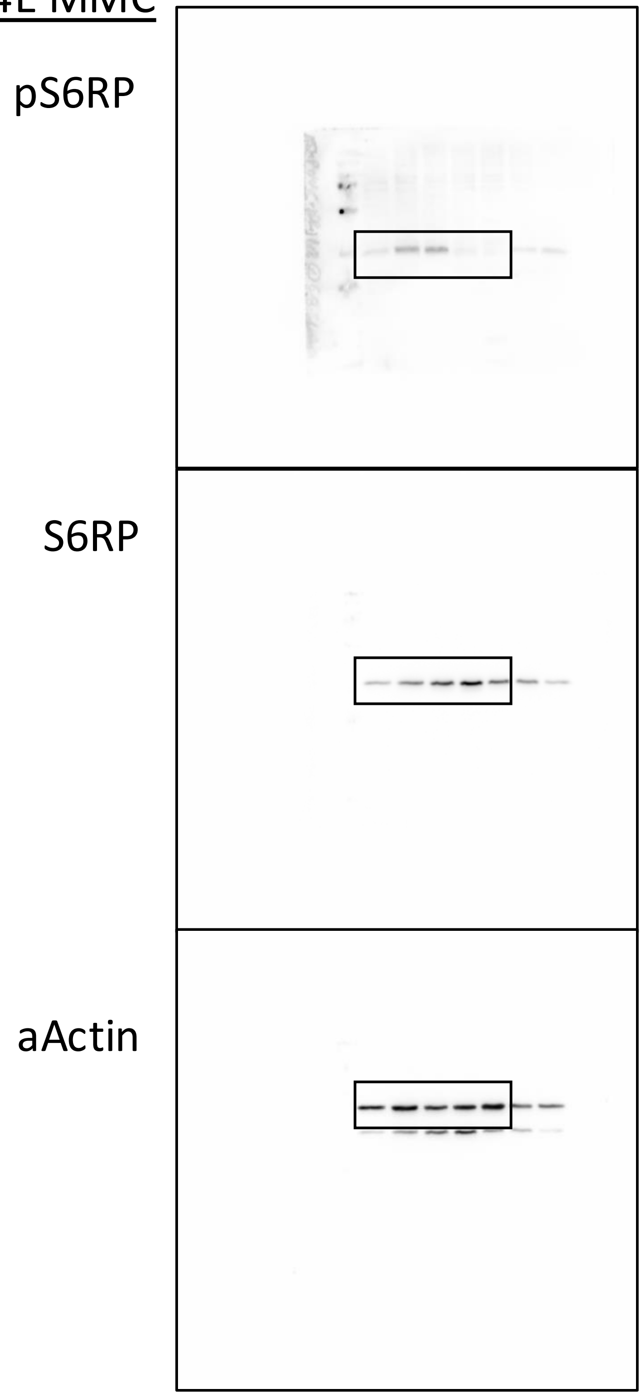

Fig. 4E UV

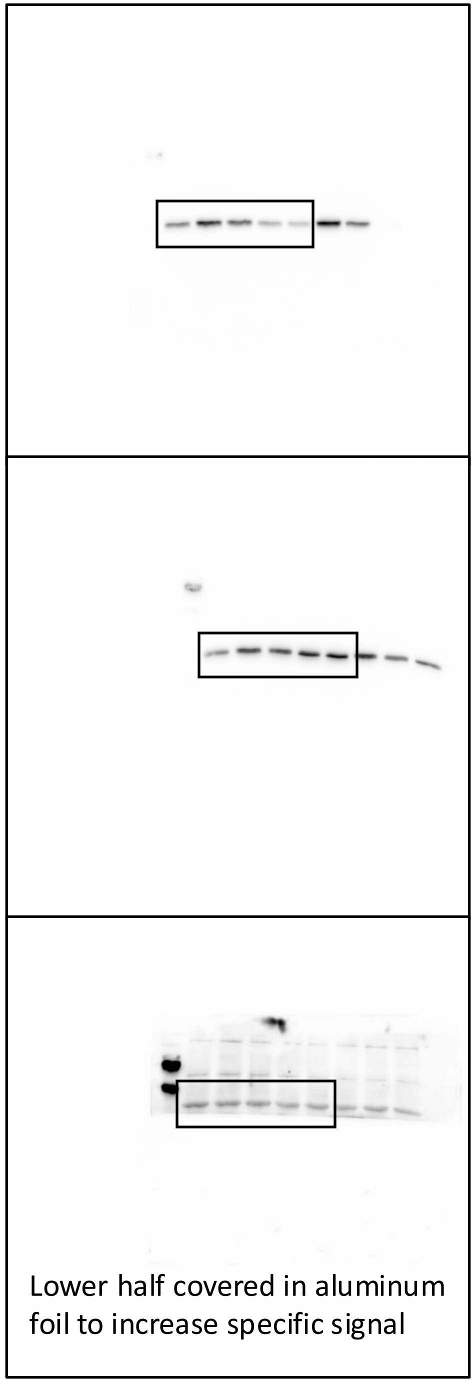

Supplement: Unedited blot and gel images [file jciinsight-10-172370-s067.pdf]
